# Development of an Infant Air-Jet Dry Powder Aerosol Delivery System (iDP-ADS) Including a New Multifunctional Bifurcating Two-Prong Nasal Interface

**DOI:** 10.1007/s11095-024-03814-y

**Published:** 2025-02-10

**Authors:** Sarah C. Strickler, Dale R. Farkas, Mohammad A. M. Momin, Laura Vargas, Ghali Aladwani, Michael Hindle, Worth Longest

**Affiliations:** 1 Department of Mechanical and Nuclear Engineering, Virginia Commonwealth University, 401 West Main Street, P.O. Box 843015, Richmond, VA 23284-3015 USA; 2 Department of Pharmaceutics, Virginia Commonwealth University, Richmond, VA USA

**Keywords:** active dry powder inhaler, air-jet dry powder inhaler, dry powder aerosol, infant aerosol delivery, nose-to-lung aerosol delivery

## Abstract

**Purpose:**

To improve the quality of aerosol delivery to infants, the iDP-ADS was advanced to include dual-prong nose-to-lung aerosol administration with a bifurcating interface, consistently monitor lung pressures and control ventilatory parameters with a pressure monitoring and control (PMC) unit, and implement flexible nasal prongs for use across a range of subject sizes.

**Methods:**

Four bifurcating flow pathways were integrated into the iDP-ADS and tested *in vitro* with a full-term infant nose-throat (NT) model for comparison to the performance of a single-prong interface. After selecting the best-performing flow pathway, flexible prong designs were evaluated in the same model and chosen for additional testing. Realistic pulmonary mechanics (PM) and age-appropriate tidal volumes were used to simulate ventilation with the PMC unit and aerosol delivery in full-term and 34-week gestational age preterm NT models.

**Results:**

Three of the four bifurcating flow pathways matched the performance of the single-prong design (tracheal filter delivery of ~55%), and the FP4 design with co-flow was selected. A flexible prong version of FP4 produced similar performance to the rigid version. Measurements from the PMC unit demonstrated that consistent air volumes under safe operating pressures could be delivered with a PEEP between 4–6 cmH_2_O. Considering aerosol delivery, PM conditions resulted in ~4% decrease in filter deposition but high lung delivery efficiencies of ~45% and ~34% for the full-term and preterm models, respectively.

**Conclusions:**

The best-performing interface with flexible prongs matched the lung delivery efficiency of a high-transmission single-prong interface and delivered high aerosol doses through late-preterm to full-term NT models.

## Introduction

Pharmaceutical aerosols have been envisioned to treat a number of infant respiratory diseases and disorders including respiratory distress syndrome (RDS), acute lung injury, viral and bacterial pneumonias, respiratory syncytial virus infection and bronchiolitis [1–5]. Despite the potential advantages of aerosol therapies, including improved efficacy with minimal side effects if properly delivered [6], very few pharmaceutical aerosol products for infants have reached the market [7–9], potentially due to challenges associated with administering pharmaceutical aerosols to infants. Regardless of the mode of inhalation aerosol delivery, challenges associated with infant subjects [4, 7, 10] include poor lung delivery efficiency (typically in the range of 1% of initial dose [11–14]), high intersubject variability [15, 16], and long delivery times for high dose medications [17, 18] (which typically are required for most envisioned infant applications, except for inhaled corticosteroids and bronchodilators).

Aerosol delivery options for infants are nebulizers, soft mist inhalers, metered dose inhalers (MDIs), and dry powder inhalers (DPIs) [5, 19], with DPI use being the least common. Compared with other modes of aerosol delivery, dry powder formulations offer the potential for improved stability [20], rapid administration [21], low cost delivery devices [22], high dose delivery [23] and potential high-efficiency lung delivery [24] with the possibility for inclusion of controlled condensational growth technology [25, 26]. Despite these potential advantages, dry powder aerosol delivery to infants has often been overlooked as most current commercial products, which are typically designed for adults, require active inhalation (i.e., resulting in a passive DPI) to form the aerosol, with inhaled volumes in the range of 3 – 4 L of air [27] (compared with the typical infant tidal volume of ~6 – 7 mL/kg [28, 29]). As reviewed by Longest *et al*. [19], active positive-pressure DPIs can be used with infants to efficiently form a small particle aerosol and provide a full inhalation breath. Previous studies that have implemented positive-pressure DPIs for infants have typically reported very low lung delivery efficiencies (e.g., <5% of loaded dose [30]); however, device performance has been improving. For example, in a recent *in vitro* and animal *in vivo* study by Walther *et al*. [31], lung delivery efficiency of ~53% was reported; however, each actuation required an air volume of 30 mL, which is high for the preterm patient population, and up to 400 hand-actuations were needed to administer a full/maximum dose. In the NICU setting, this high number of actuations would require significant time and effort from the clinicians. The development of a positive-pressure DPI system that can solve the current challenges associated with administering pharmaceutical aerosols to infants could potentially open the door to additional commercial products (such as an aerosolized surfactant therapy (AST) for RDS) with improved efficacy, thereby improving treatment outcomes for a number of infant respiratory diseases and disorders beyond asthma.

Our group has recently developed a positive-pressure DPI for high-efficiency aerosol generation and delivery with very small air actuation volumes (e.g., 3 – 10 mL) [21, 23, 24, 32–34]. Potential applications of these air-jet DPIs include aerosol generation and delivery during non-invasive ventilation, nose-to-lung aerosol delivery, as well as aerosol delivery to infants, young children, and test animals [35, 36]. Given that infants are obligate nasal breathers, unless under certain conditions such as nasal occlusion, the nasal route was selected for aerosol administration [13, 37]. An infant air-jet DPI was recently developed for efficient aerosol delivery simultaneous with non-invasive ventilation (NIV), e.g., during nasal continuous positive airway pressure (CPAP), or directly through the nose to the lungs of an infant (direct-to-infant; D2I) using a nasal prong interface [38–41]. Key components of the infant air-jet DPI include a small diameter inlet airflow passage, aerosolization chamber, outlet flow passage, and patient interface (including a diffusional flow pathway and nasal prong(s)) [21]. As previously described, small boluses of air are actuated into the small diameter inlet and enter the aerosolization chamber at high velocity [32, 42]. Air-jet expansion within the aerosolization chamber forms the aerosol via secondary velocity currents. The outlet flow passage (often a metal capillary) serves as a size selection filter allowing sufficiently deaggregated particles to escape. A diffusional flow passage is then a key element that is required to slow the outlet aerosol flow (diffusing the turbulence) with a minimal amount of aerosol depositional loss [39]. Similarly, the prong section of the patient interface can create depositional loss and impact transmission of the aerosol through the nose [39, 43]. Development of the infant air-jet DPI thus far has primarily focused on the D2I approach, which has the advantage of not requiring compatibility with a specific form of NIV or a manufacturer’s ventilation circuit and/or interface and can administer an aerosol either prior to NIV support or during a brief period of NIV removal, as occurs with regular interface repositioning and cleaning.

Taken together, these individual components developed in previous studies comprise the infant air-jet dry powder aerosol delivery system (iDP-ADS). Components previously considered for sensitivity analysis and optimization have included the aerosolization engine (inlet and outlet flow passages and aerosolization chamber) [21, 38], positive-pressure air source [40], and patient interface selection [39]. Based on this previous development, *in vitro* testing of lung delivery efficiency through representative full-term (~3500 g) and preterm (1500 – 1600 g) infant nasal models have indicated that 50 – 60% of initially loaded spray-dried formulations reach a tracheal filter, and this lung delivery efficiency is not impacted by the presence of downstream pulmonary mechanics (PM) [38–40, 44]. The rate of aerosol delivery is also rapid and controllable, with ~5 mg of powder formulation delivered per breath [44].

During previous iDP-ADS development and testing, several important observations related to the patient interface have been made. First, use of an aerosolization chamber with two outlets leading to a dual nasal prong design increases nasal depositional loss by ~10% absolute difference and reduces lung delivery efficiency of the aerosol by 10 – 15% (absolute difference), compared with a single-prong design [39]. Secondly, Bass *et al*. [43] implemented computational fluid dynamics (CFD) simulations to demonstrate that the presence of infant nasal prongs increases nasal depositional loss of inhaled aerosol by 20 – 70% (absolute difference) compared with nasal deposition during inhalation without prongs. Hence, the interaction of the prongs and nasal region has significant potential to improve the lung transmission of aerosol. Thirdly, Bass *et al*. [43] theoretically demonstrated that a two prong nasal configuration has the potential to reduce nasal depositional loss by spreading the delivered flow across both nostrils, which provides a reduced aerosol Stokes number [45, 46].

While previous development of the iDP-ADS has indicated efficient and rapid lung delivery of therapeutic aerosols, a primary limitation is the use of a single nasal prong for D2I aerosol administration. This approach requires implementing the resuscitation technique of te Pas and Walther [47], in which the nasal prong is inserted into one nostril and the contralateral nostril is held closed by the administrator during device actuation and then opened to enable exhalation. This approach will likely require significant skill and training of the clinician operator and interaction with the infant. A bifurcating interface with two nasal prongs may be more convenient, require less interaction with the infant, enable better pressure monitoring and positive end expiratory pressure (PEEP) support, and further improve lung delivery of the aerosol. However, development of a bifurcating system will require analysis of the diffusional flow pathway and prongs to minimize aerosol depositional loss. Furthermore, use of a bifurcating interface during D2I aerosol delivery with high dose medications will require that the interface be left in place for multiple actuations (e.g., ~10 breath cycles for a 50 mg dose). An exhalation port will therefore need to be included, similar to that of a conventional T-piece (NeoPuff^®^) device that is operated by the administering clinician. A pressure gauge is needed to monitor maximum pressure (positive inspiratory pressure; PIP) and to help maintain PEEP within the lungs. Sealing between the prongs and the nostrils is also critical to minimize aerosol and pressure losses [48]. The final prong design will need to be flexible and include an innovative tip for use across different nostril dimensions (diameters and spacing).

The objective of this study was to develop a new iDP-ADS with a bifurcating patient interface for D2I aerosol administration that: (i) either matches or surpasses the lung delivery efficiency of a single-prong interface, (ii) enables controlled exhalation and monitors airway pressure, and (iii) can accommodate different nasal dimensions and widths with one device. Key system components include the positive-pressure air source (electromechanical (EM) timer) [40], aerosolization engine [39], new bifurcation interface, and recently introduced pressure monitoring and control (PMC) unit [34]. Multiple bifurcating flow pathways, including an innovative co-flow design, were first explored to identify a design that best maintains high lung delivery efficiency. Use of different nasal prong geometries (component that is in closest contact with the nostrils) were also explored. A PMC unit [34] that is responsible for (i) monitoring administered pressure, (ii) enabling exhalation with resistance (to better maintain PEEP), and (iii) giving the operator better control of the administered pressure range was applied to a non-invasive aerosol delivery strategy in infant models. System performance was initially based on aerosol administration through a realistic *in vitro* nose-throat (NT) geometry representative of a full-term infant. While envisioned applications include administration of aerosolized antibiotics, surfactants, antivirals, and vaccines, design development initially implemented a model spray-dried excipient enhanced growth (EEG) formulation [49] containing albuterol sulfate (AS) as a readily quantifiable drug. After selecting the best flow passage and nasal prong design, additional testing with the PMC unit implemented realistic downstream lung mechanics (compliance and resistance) mimicking an infant with RDS who has surfactant-deficient lungs [50] and also explored use in a preterm nasal geometry.

## Materials and Methods

### Phase 1: Experimental Overview, Setup, and Components

**Fig. 1:**
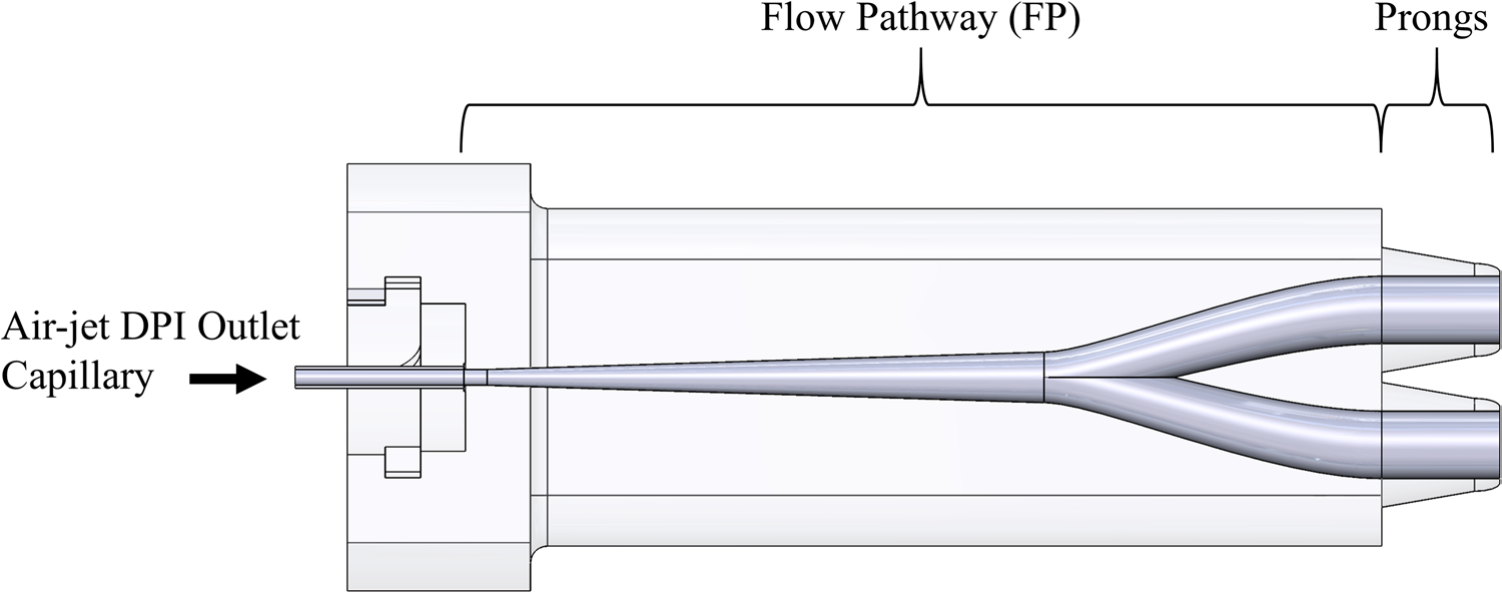
Overview of the patient interface components. The air-jet DPI outlet capillary delivers the aerosol stream to the flow pathway (FP), which diffuses the turbulent jet. The flow pathway also includes the bifurcation for dual-outlet interfaces. The aerosol then passes through the prongs, which are inserted and oriented within the nostrils for introduction of the aerosol into the nasal cavity.

Phase 1 involved *in vitro* testing of the patient interface in a 3D-printed anatomically-correct full-term NT model using an albuterol sulfate excipient enhanced growth (AS-EEG) formulation for the model drug. The interface is comprised of the diffusional flow pathway and the prongs, which are partially inserted into the nostrils (Fig. 1). The flow pathway extends from the air-jet DPI outlet capillary until the prongs and includes the bifurcation geometry. Flow pathways and prongs were examined separately to understand their effects on aerosol transmission. Following preliminary *in vitro* screening, four flow pathways (referenced from here onwards as “FP*x*”) with bifurcations and a base prong geometry were designed and tested against a baseline single-outlet interface (FP0). The best bifurcating flow pathway in terms of delivered lung dose was chosen and used for testing prong designs for comparison with the base case prongs. Important considerations of this aerosol delivery platform were to deliver the full inhalation breath and to ensure an airtight seal between the prongs and the infant’s nostrils to prevent aerosol leakage and help gently expand the infant’s airways with the positive pressure created by the delivered air volume. These components helped to produce consistency and reproducibility during aerosol delivery. As a result, prong designs with high aerosol transmission efficiencies that can also provide adequate sealing with a range of infant nostril geometries and sizes were sought.

**Fig. 2:**
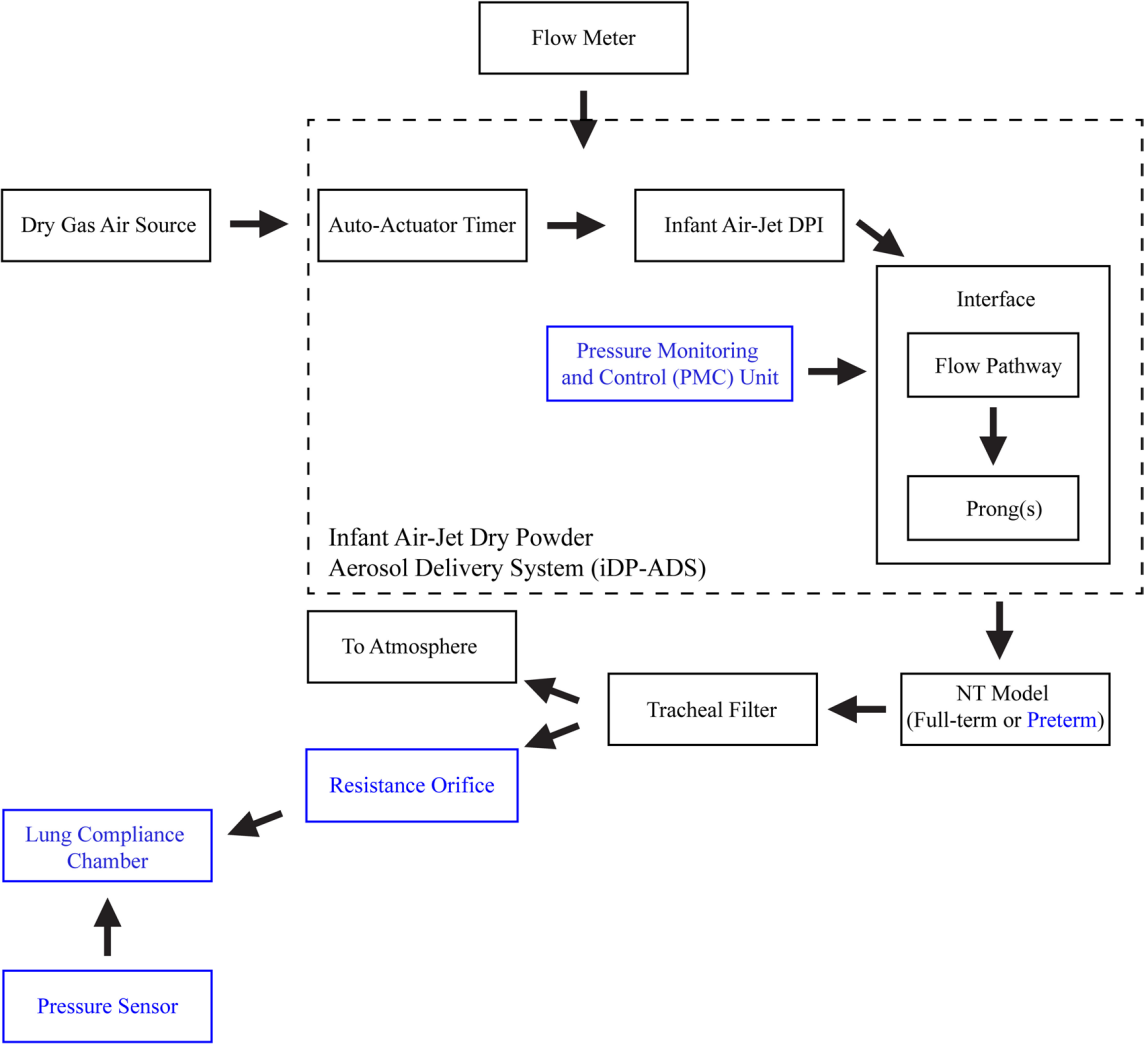
Schematic of the experimental setup for assessing the delivered lung dose (approximated by the Tracheal Filter dose) and deposition in the Infant Air-Jet Dry Powder Aerosol Delivery System (iDP-ADS) and Nose-Throat (NT) Model when the filter is open to the atmosphere and when realistic infant pulmonary mechanics are simulated (blue).

**Fig. 3:**
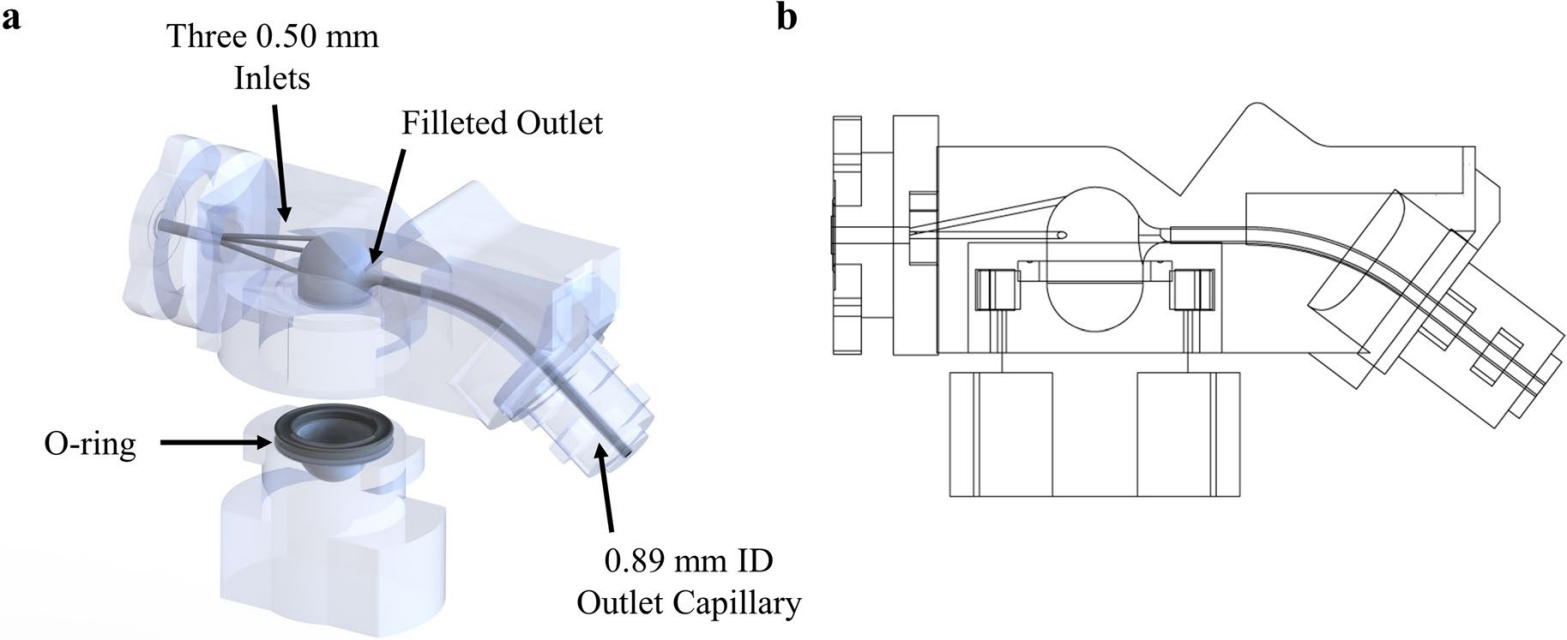
Infant air-jet DPI. (**a**) Exploded isometric view showing the components of the DPI and demonstrating how the aerosolization chamber is opened for powder loading. (**b**) Side view of the fully assembled device depicting the vertical aerosolization chamber design. (ID = inner diameter).

**Fig. 4:**
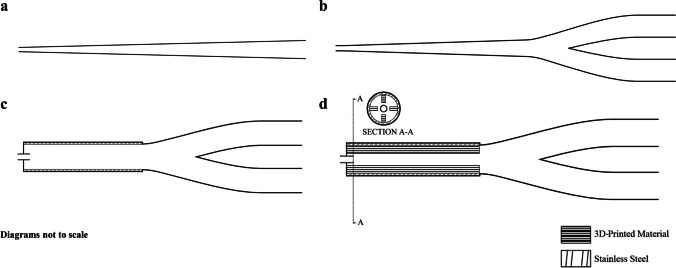
(**a**) FP0 or baseline gradual expansion single-outlet interface. (**b**) FP1 or gradual expansion dual-outlet interface. (**c**) FP2 or rapid expansion dual-outlet interface with overhanging inlet capillary. (**d**) FP3 or rapid expansion dual-outlet interface with four 3D-printed fins oriented along the longitudinal axis.

**Fig. 5:**
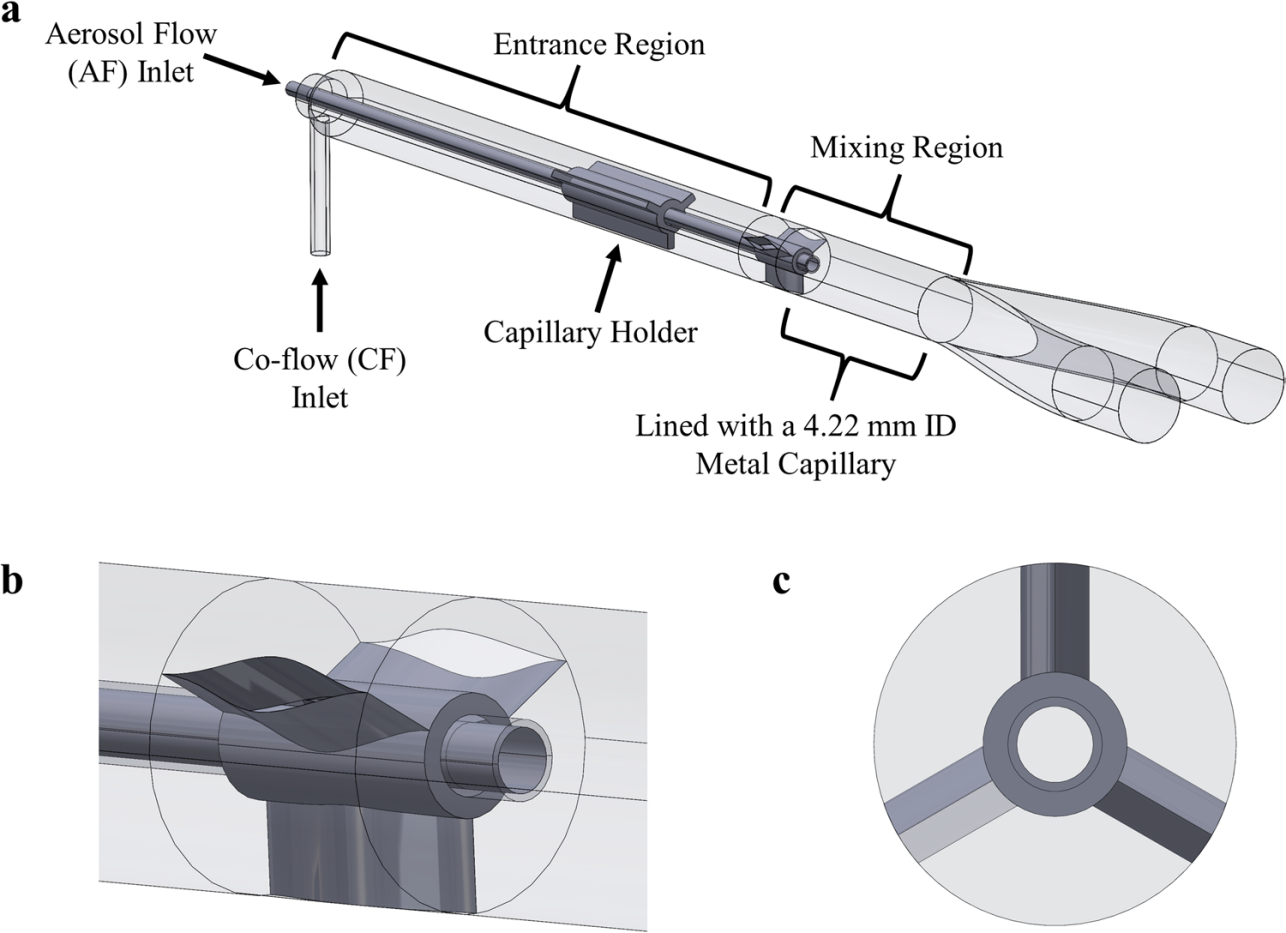
FP4 or co-flow interface design. (**a**) Isometric view showing the co-flow (CF) inlet and the metal capillary aerosol flow (AF) inlet as well as the entrance and mixing regions. (**b**) Close up view of the region where the capillary enters the rapid expansion region (beginning of the mixing region) showing the symmetrical airfoil-inspired design to help axially align the capillary while also minimizing boundary layer separation and maximizing the open area for incoming CF. (**c**) Axial view of the mixing region. (ID = inner diameter).

**Fig. 6:**
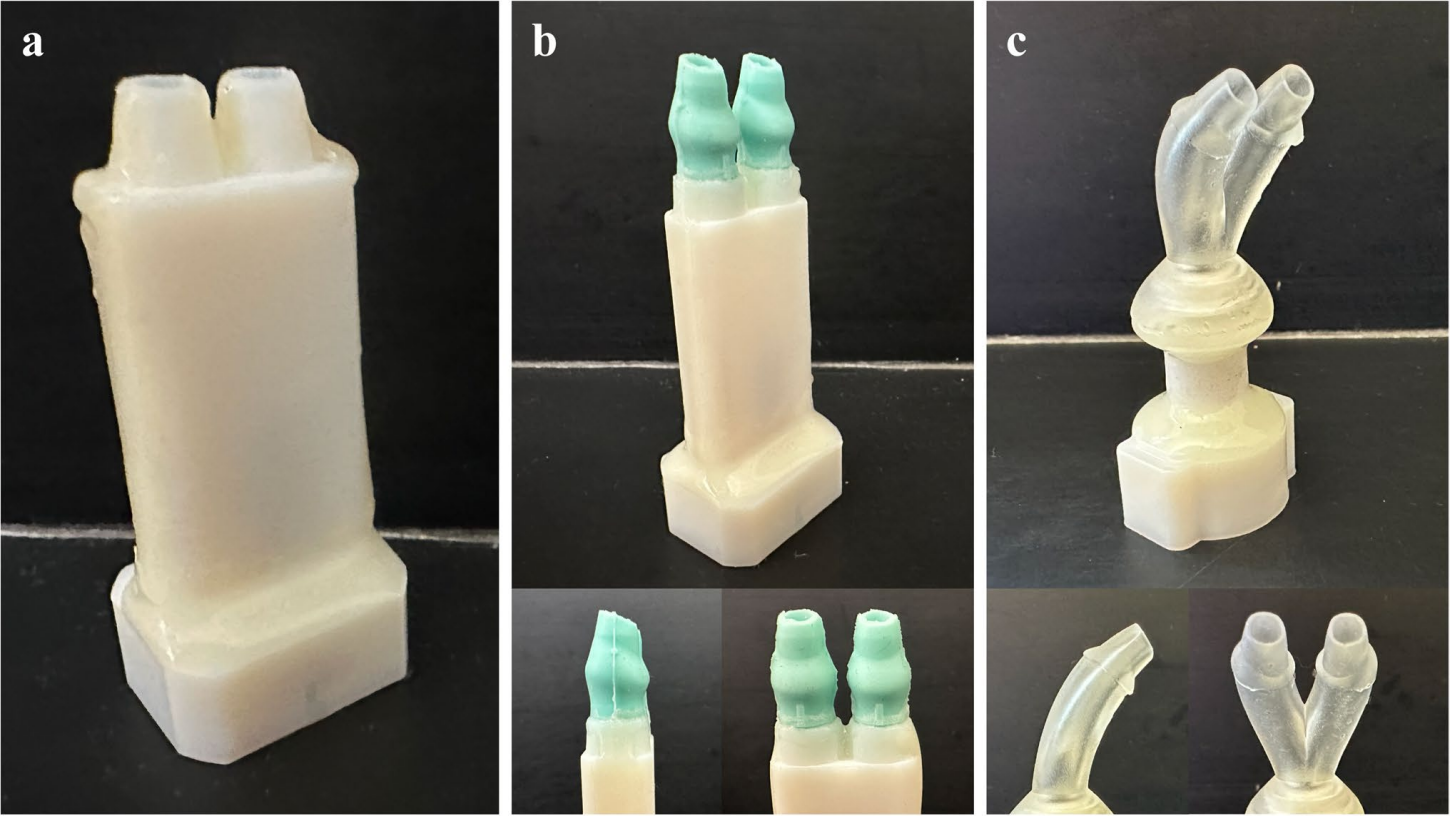
(**a**) 3D-printed interface with rigid conical prongs. (**b**) Silicone-casted flexible prongs (Flex1) with angled cuts at the outlets and expanded nasal pillows. (**c**) Curved prongs and bifurcation 3D-printed with flexible material (Flex2).

**Fig. 7:**
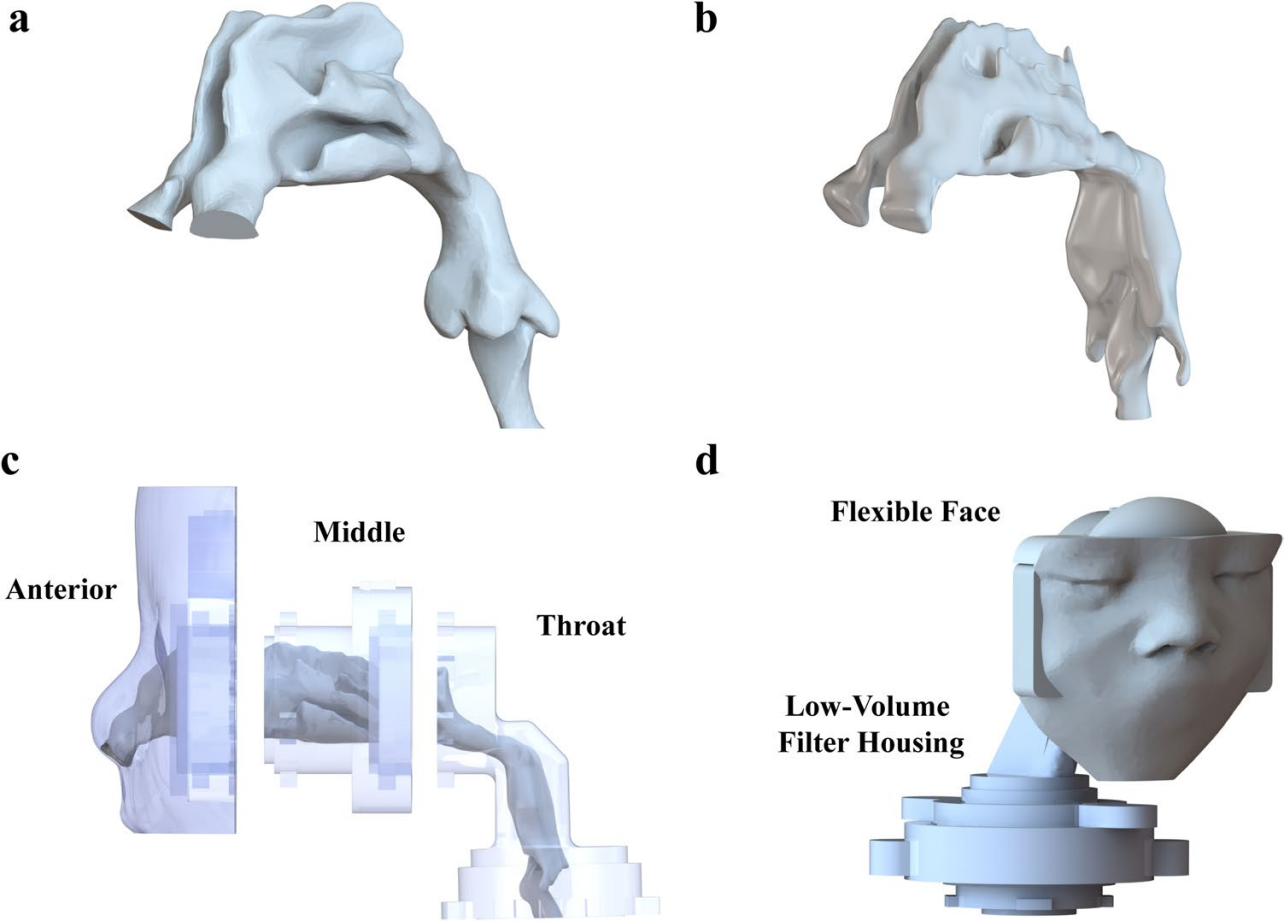
Airways of the (**a**) full-term (3.55 kg) and (**b**) preterm (2.20 kg) nose-throat (NT) models. (**c**) Definition of regional zones shown using the sectioned 3D-printed preterm NT model. (**d**) Flexible face, NT model, and low-volume filter housing for the 3D-printed full-term model.

The Phase 1 experimental setup (Fig. 2, in black) consisted of a compressed dry-air source to minimize hygroscopic growth of the formulation, the auto-actuator (electromechanical (EM)) timer [40], a Sensirion neonatal mass flow meter (Sensirion SFM3400, Sensirion AG, Stafa, Switzerland), the infant air-jet DPI (Fig. 3), the nasal interface tested with different flow pathway (Figs. 4 and 5) and prong configurations (Fig. 6), the full-term NT model (Fig. 7a) [38], and a filter with a low 2.7-mL filter housing volume prior to the filter material (Fig. 7d). This low-volume (LV) filter was designed to minimize dead space given the low actuation volumes required for the neonatal patient population [40]. The infant air-jet dry powder aerosol delivery system (iDP-ADS), which consists of the auto-actuator timer, infant air-jet DPI, and nasal interface, was designed to deliver high lung doses of powder formulation (10 – 100+ mg) with low gas actuation volumes (10 – 30 mL) and flow rates (1.7 – 4.0 LPM). The auto-actuator supplied the external energy source needed to disperse the powder and consistently controlled the flow rate and actuation time across multiple actuations using an electric timer and solenoid valves, creating a square inhalation flow profile [40]. The infant air-jet DPI (aerosolization engine, Fig. 3) used in this study was the D1 device described in Howe *et al*. [39, 40]. Briefly, it has three 0.5 mm inlet channels, a 0.28 mL vertical aerosolization chamber, and a filleted 0.89 mm outlet connecting to a stainless steel (SAE 304) capillary that gently curves 37° before reaching the interface. This curved capillary allowed the device to be held horizontally so that the aerosolization chamber, which opened to load powder (Fig. 3a), remained level under the device. Twist lock connections with O-rings were located in the aerosolization chamber and at the connections between the flow meter and device and between the device and interface to provide airtight connections. In other studies, we have developed designs in which this air-jet infant DPI can be cyclically reloaded after each actuation without operator intervention using gravity filling of the aerosolization chamber for high dose applications such as during aerosol surfactant therapy (AST) [44, 51].

The infant air-jet DPI, flow meter adaptors, and patient interfaces were designed in SolidWorks (Dassault Systèmes, Paris, France) and exported as STL files. The DPI, adaptors, and rigid patient interface pieces were printed on a Stratasys Objet24 3D Printer (Stratasys Ltd., Eden Prairie, MN) using VeroWhitePlus resin and a resolution of 32 µm. All surfaces contacting the powder were printed in glossy mode for a smoother finish to minimize powder retention. Construction of the flexible prongs is discussed in the *Flexible Prongs* subsection.

For each experiment in Phase 1, the device was loaded with a nominal dose of 10 mg of the model AS-EEG formulation and actuated with a nominal 30 mL volume of air, consistent with a tidal volume of <10 mL/kg for a full-term neonatal model setup [29, 38, 52]. The actuation flow rate was set using the Q90 metric, which represents the flow rate at which 90% of the flow rate measurements fall below. The Q90 metric has been a convenient parameter that translates well between different flow rate profiles and air sources [40].

### Flow Pathways

The flow pathways (Fig. 4), as described below, were designed based on several different physical principles but shared common features, such as their bifurcation and prongs, during flow pathway testing. The bifurcation (for the dual-outlet designs) was streamlined [53, 54] using a minimum radius of curvature of ~4 mm along the bifurcation’s centerline to minimize deposition from impaction and gently guide the airflow. A rigid conical prong with an outlet inner diameter (ID) of 4 mm was used for both single- and dual-outlet flow pathways.

#### FP0 – Gradual Expansion with Single-Outlet

Flow pathway 0 (FP0), or the single-outlet gradual expansion interface (Fig. 4a), has been our previous best-performing design [39]; therefore, it was used as the baseline interface in this study. As described in our previous work for delivery to a full-term infant [38], the inlet and outlet diameters were 0.89 mm and 4 mm, respectively. The 63-mm length provided a slow (gradual) increase in diameter to prevent separation of the boundary layer, minimize deposition, and diffuse the velocity of the aerosol jet prior to the prong and nose.

#### FP1 – Gradual Expansion with Bifurcation

Flow pathway 1 (FP1), or the dual-outlet gradual expansion (Fig. 4b), leveraged the benefits of FP0 but added the streamlined bifurcation to the outlet. The inlet and outlet diameters for the gradual expansion region were 0.89 mm and 3 mm, respectively. The smaller increase in diameter compared to the FP0 interface permitted a shorter expansion region of 33 mm and helped to minimize dead space (i.e., the volume of gas that will not reach the lungs and remains in the interface or extrathoracic airways). Connected to the 3-mm outlet was a bifurcation that expanded to two 4-mm outlet prongs over a 20-mm length. The center-to-center separation distance between the two prong outlets was 8 mm. To help straighten the flow before entering the nasal cavity, a 7-mm cylindrical extension was added, resulting in a total interface length of 60 mm, similar to the FP0’s length. During preliminary testing, these dimensions of the dual-outlet gradual expansion performed the best in terms of filter delivery.

#### FP2 – Rapid Expansion with Bifurcation

Flow pathway 2 (FP2), or the rapid expansion interface (Fig. 4c), consisted of a 20-mm long, 4.22-mm ID stainless steel (SAE 304) capillary with a similar bifurcation as FP1. The expansion region gave the incoming aerosol jet space to diffuse and has been shown using CFD to reduce aerosol losses in interfaces for the pediatric patient population [55]. The outlet capillary from the device was positioned in the center of the larger rapid expansion metal capillary and extended 1 mm to overhang inside the larger capillary. The surface roughness of stainless steel is less than the 3D-printed material; therefore, it was hypothesized that particle adhesion and thus interface losses would be minimized.

#### FP3 – Rapid Expansion with Fins and Bifurcation

Flow pathway 3 (FP3), or the rapid expansion interface with fins (Fig. 4d), was identical in dimension to FP3 but included four 3D-printed 0.60-mm thick fins positioned 90° from each other that protruded 1.20 mm into the central flow passage from the inner wall of the rapid expansion capillary. These fins were included to help slow and diffuse the incoming high-velocity aerosol jet and prevent oscillating jet flow arising from the time-varying Coandă effect as seen in previous CFD simulations [55].

#### FP4 – Co-flow with Bifurcation

Flow pathway 4 (FP4), or the co-flow interface (Fig. 5), required two flow streams, aerosol flow (AF) and clean air co-flow (CF), and consisted of two components, the entrance region and the mixing region. The entrance region on the left of Fig. 5a was where the AF entered from the device and continued in an extended capillary into the mixing region on the right. The CF made a 90° turn and expanded to fill the 4.22-mm ID of the entrance region surrounding the AF capillary. The beginning of the mixing region (Fig. 5b) had three evenly-spaced supports (Fig. 5c) to hold the AF capillary along the central axis. These supports had an air-foil inspired design that sought to minimize separation of the boundary layer from the supporting structure to achieve a uniformly-distributed velocity profile as the CF entered the mixing region. The CF was introduced into the mixing region using the largest area possible to create a thick air buffer into which the incoming AF could diffuse. For experimental testing, the entrance and mixing regions were split into two different parts to allow analysis of individual components; therefore, another capillary holder (Fig. 5a) was placed in the entrance region to aid alignment of the capillary during assembly. These supports were rectangular prism supports. The mixing region had an identical design as FP2 (Fig. 4c) with the AF capillary overhanging 1 mm into the larger metal-lined rapid expansion. The addition of CF had multiple potential benefits. First, the CF should provide an air sheath that protects the AF from the walls of the interface, thereby minimizing interface losses. Second, the CF should stabilize and diffuse the AF jet, perhaps leading to improved flow introduction to the anterior nose in order to reduce depositional losses.

To realize these benefits, the flow parameters, not just the geometry, had an impact on performance. For the other FPs, the following Q90s were used: 1.7 LPM for the FP0 and 2 LPM for FP1 – 3. (In preliminary testing, using 1.7 LPM or 2 LPM for the bifurcating interfaces did not affect deposition measurements as it did for the single-prong interfaces, resulting in a significant improvement in filter delivery at 1.7 LPM as compared to 2 LPM [40]). Table I shows the delivery parameters and flow metrics for the AF and CF streams for FP4. In determining the appropriate parameters, there were several considerations. First, the actuation time should be left constant between the two streams to prevent adding the additional factor of chase air and, hence, isolate the effect of CF alone. Second, a majority of the flow should be used for CF to increase the amount and force of the air sheathing the aerosol. Third, the flow rate and actuation volume for creating the AF should be large enough to ensure adequate aerosolization. For this air-jet DPI, the lower limit for the Q90 was 1.7 LPM, which was also consistent with the aerosolization flow rate used for testing FP0. Previous work with similar versions of low-volume DPIs aerosolizing AS-EEG powders have shown similar mass median aerodynamic diameter (MMAD) and device emptying performance between 10 mL and 30 mL actuation volumes [38]. Choosing the 10 mL actuation volume and a Q90 of 1.7 LPM for the AF resulted in 20 mL and 3.4 LPM for the CF actuation volume and Q90, respectively, for identical actuation times of 0.35 s. As shown in Table I, the ratios of CF-to-AF for Q90s and actuation volumes were both 2. Because of the large volume of the CF entry region, the velocity of the CF flow was relatively low (~4 m/s) and the Reynolds number was well below laminar conditions (~800), indicating a relatively stable CF stream.

**Table I:** FP4 (Co-Flow Interface Design) Delivery Parameters for the CF and AF Streams

Parameter	Nominal Actuation Volume (mL)	Q90 (LPM)	Actuation Time (s)	Average Velocity* (m/s)	Reynolds Number*
AF	10	1.7	0.35	45.5	2682
CF	20	3.4	0.35	4.05**	809
Total Flow Stream	30	5.1	n/a	n/a	n/a
Ratio (CF/AF)	2	2	1	0.14	0.30

^*^Theoretical, based on initial design dimensions

^**^At the point where the CF enters the AF-CF mixing region after the fins

AF = Aerosol flow, CF = Co-flow

### Flexible Prongs

To replace the rigid prongs (Fig. 6a) used during flow pathway testing, flexible prongs were designed to aid sealing within the nostril across a wide patient population, potentially improve patient comfort, and reduce chances of nasal injury. Two flexible prong designs (Fig. 6b, c) were tested. The first prong design, Flex1 (Fig. 6b), was cast in platinum-cure 60 Shore A silicone (Smooth-Sil™ 960; Smooth-On, Macungie, PA) in molds printed at a 50-µm resolution on a Formlabs Form 3+ stereolithography (SLA) 3D printer (Formlabs, Boston, MA) in Clear Resin V4. The prongs added a ~20-mm, straight section post bifurcation outlet. The center-to-center separation distance between the prongs was increased from 8 mm to 10 mm to accommodate a larger range of septal distances. The prongs included an angled cut with a small wedge to ease insertion, which required that the internal diameter of the bifurcation outlet and prong be 3.6 mm for a sufficient casting thickness at the tip (~0.6 – 0.7 mm). An external nasal pillow helped to seal the nostril and control insertion depth.

The second design, Flex2 (Fig. 6c), included an entirely flexible bifurcation and prong that was 3D-printed with Formlabs’s Flexible 80A resin (80 Shore A). This pliable bifurcation could help further increase the allowable variability in patient septal distances and permit better alignment of the interface with patient-specific nostril anatomy. The bifurcation included a 45° curve with a radius of curvature of 25 mm to minimize impaction as the particles change direction. In previous ventilator component and cannula streamlining work, a radius of curvature of at least 10 mm greatly decreased depositional losses by producing a more uniform flow [53, 56]. Curving the bifurcation was investigated to see if future DPI designs could be held level without including the more complex manufacturing process of bending a capillary. The prong portion was angled 5° inward and curved downwards 15° with a 25-mm radius of curvature to match the bifurcation flow pathway. The prongs were not cut at an angle at the ends as in the Flex1 design, but did have IDs of 3.6 mm. Additionally, the cone comprising the nasal pillow was angled inward and had a noncircular cross section with an elbow on the outer bottom corner to assist sealing at the lateral nostrils, which were typically most susceptible to air leaks. In total, the length of the bifurcation and prong was ~27 mm, which was significantly shorter than the ~40 mm for the previous flexible prong version, thereby reducing dead space. All prongs, including the rigid baseline prongs, were designed to be inserted ~5 – 7 mm into the nostrils.

### Phase 2: Pulmonary Mechanics Experiments

**Fig. 8:**
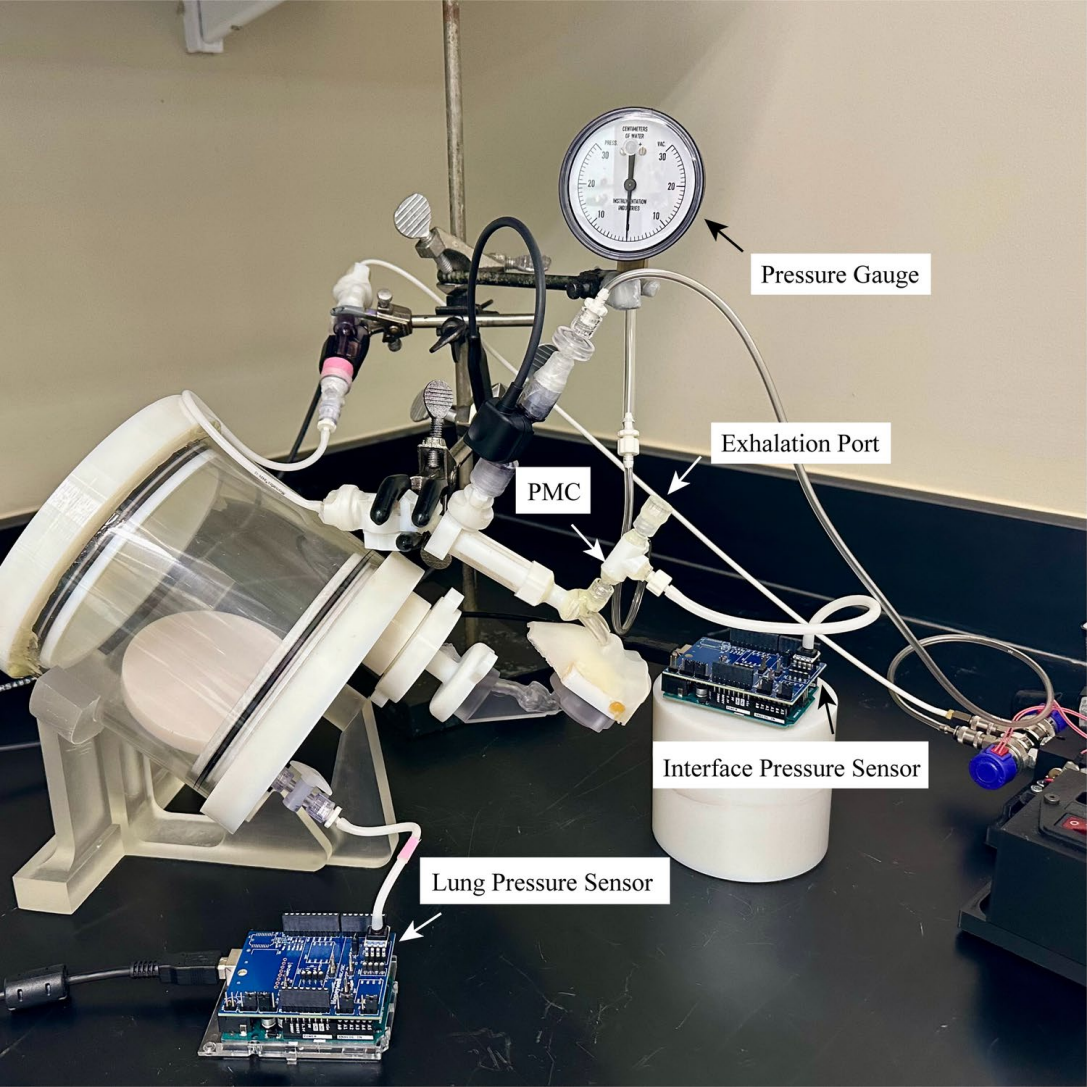
Pulmonary mechanics setup (full-term shown) displaying the pressure monitoring and control (PMC) unit with pressure gauge (for convenient pressure monitoring during aerosol administration) and sensor (for precise recorded pressure measurements for later analysis) and exhalation port, which is covered by the operator’s thumb during inhalations and breath-holds. A lung pressure sensor was included in the compliance chamber to verify safe operating pressures.

Phase 2 of the experiments tested the best-performing flow pathway and prong combination in the full-term (3.55 kg) infant NT model and a 34-week gestational age (GA) preterm (2.20 kg) infant NT model (Fig. 7) with size-specific pulmonary mechanics (PM) setups and breathing parameters. The experimental setup (Fig. 2, in blue) included the addition of a pressure monitoring and control (PMC) unit [34] at the interface, a resistance orifice after the filter to approximate resistance of the lung, an airtight lung compliance chamber, and a pressure sensor (SSCDLNN040MGSA5, Honeywell, Sensing and Control, Golden Valley, MN) to monitor the approximated lung pressure in the chamber (Fig. 8). A similar setup has been used previously to model PM downstream of an airway model [40].

Barotrauma, volutrauma, and atelectrauma are serious complications of improperly-applied ventilation and can lead to ventilator-induced lung injury and chronic lung diseases [57–59]; thus, pressure monitoring and proper selection of aerosol delivery parameters are essential for protecting the fragile infant lung and optimizing patient ventilation. Herein lies the tradeoff: sufficient volumes and pressures are needed to prevent atelectasis (cyclic collapse of alveoli), but excessive ventilation yields injury. To ensure airway pressures during aerosol delivery do not harm the respiratory tract, pressure measurements were acquired at both the interface and the lung compliance chamber. To accomplish monitoring at the patient interface and breathing control, the PMC unit [34] was used and consisted of a pressure sensor (same model as the pressure sensor in the compliance chamber), a pressure gauge for guiding the iDP-ADS operator (Instrumentation Industries Inc., Bethel Park, PA), and a 0.5-mm exhalation orifice that could be opened or closed by the operator’s thumb and was sized to permit controllable patient exhalation (Fig. 8) during the breathing maneuver for aerosol delivery that is detailed below. The ventilation parameters implemented and monitored in this ventilation strategy included the peak inspiratory pressure (PIP), tidal volume, and positive-end expiratory pressure (PEEP). The target PIP for beginning ventilation should be between 16 – 20 cm H_2_O [60], and some sources say higher pressures of 25 cm H_2_O are safe [61]. However, since the D2I system presented here was meant to encourage deeper inhalation and supply only a handful of breaths (fewer than a resuscitator), slightly higher pressures may be acceptable. In fact, pop-off valves on resuscitation bags are typically between 30 – 40 cm H_2_O [52]. Therefore, a PIP of 25 cm H_2_O (and ideally ≤ 20 cm H_2_O) was chosen. PEEP in the range of 4 – 6 cm H_2_O is a common starting point during ventilation [60, 62]. Considering the tidal volume, typical values are around 4 – 6 mL/kg for infants with RDS who have received surfactant [63], but ventilation tidal volumes up to 7 – 8 mL/kg have been suggested [64, 65]. Tidal volume measurements averaging up to 7.3 mL/kg in infants with mild RDS (median weight of ~1 kg) have been reported [66]. Since deeper inhalation was desired with the D2I approach, a target tidal volume of 6.7 mL/kg with an acceptable range of 6.0 ± 1 mL/kg was selected; therefore, the 3.55-kg full-term infant model received a tidal volume of 23.7 mL (range: 17.8 – 24.8 mL) and the 2.20-kg preterm infant model received 14.7 mL (range: 11.0 – 15.4 mL). It should be noted though that each of these parameters is contingent on the infant’s severity of disease state.

Realistic PM values were investigated and the apparatus was calibrated for each infant model. The resistance orifice, which represented the lung resistance, allowed the model lung resistance to be tuned by changing its diameter. Calibration was performed by actuating a continuous flow at 5.1 LPM (the total flow rate for the chosen interface) through the orifice while it was open to the atmosphere. A pressure sensor placed before the orifice recorded the pressure drop. A lung resistance of 100 ± 20 cm H_2_O/L/s was targeted as it lies close to the middle of the resistance range for infants with RDS (~60 – 200 cm H_2_O/L/s) [66–68], and was 108 and 113 cm H_2_O/L/s for the full-term and preterm PM setups, respectively (Table II). The lung compliance chamber operated on the principle of the compressibility of air. By using the ideal gas law, the volume of air needed to achieve a change in pressure was calculated and used to design the compliance chamber for the desired static compliance. Compliances have a significant range across the preterm and term patient populations (~0.3 – 0.8 mL/cm H_2_O/kg for infants with RDS who have received surfactant [63]) but have typical values around 0.3 – 0.5 mL/cm H_2_O/kg for infants with RDS who have yet to receive surfactant [67, 68]. (The compliances reported in the literature are dynamic compliances; however, the compliance chamber could only model static compliance.) A compliance of 0.5 ± 0.1 mL/cm H_2_O/kg was selected to represent infants that may not require mechanical ventilation, resulting in a target compliance of 1.78 and 1.10 mL/cm H_2_O for the full-term and preterm models, respectively. The chamber was calibrated by actuating the target actuation volume into the chamber only and measuring the stabilized lung pressure. The measured compliances of 1.79 and 1.07 mL/cm H_2_O for the full-term and preterm models, respectively, were very near the targeted values (Table II).

**Table II:** Delivery Parameters and Pulmonary Mechanics for the Experiments with Positive-End Expiratory Pressure (PEEP) for Both the Full-Term and 34-Week Preterm Models

Model	Full-Term	34-Week Preterm
Target Actuation Volume (mL)	23.7	14.7
Target AF Volume (mL)	7.9	4.9
Target Compliance (mL/cm H_2_O)	1.78	1.10
Measured Compliance (mL/cm H_2_O)	1.79	1.07
Measured PIP (cm H_2_O)	~19	~22
Measured Resistance (cm H_2_O/L/s)	108	113

AF = Aerosol flow, CF = Co-flow, PIP = Peak inspiratory pressure

**Fig. 9:**
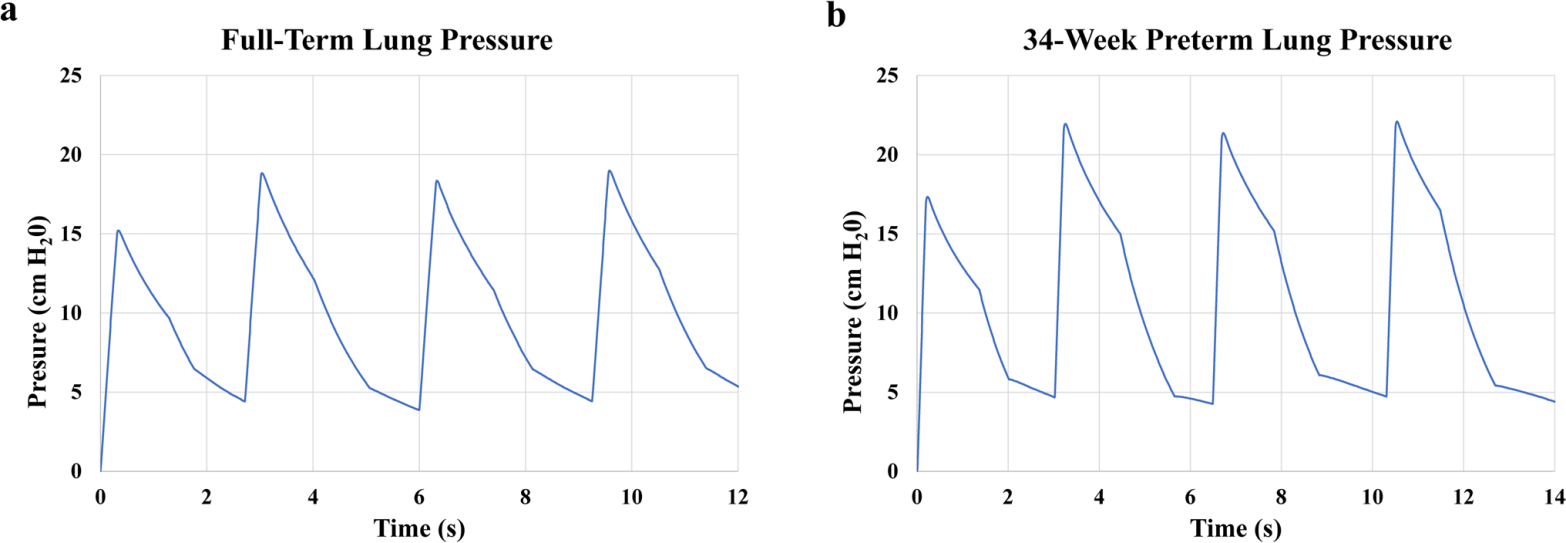
Lung pressure profiles for the (**a**) full-term and (**b**) 34-week preterm models generated using the best-performing interface (FP4 with Flex2 prongs). The cycle shows inhalation and a 1 – 2 s breath-hold while the operator’s thumb was placed over the exhalation port on the pressure monitoring and control (PMC) unit, followed by exhalation upon removal of the thumb until the desired positive-end expiratory pressure (PEEP) was reached and the thumb was replaced on the port for the next breathing cycle.

Using this PM setup with the infant NT model, the aerosol delivery and ventilation strategy was implemented and tested without powder using the full iDP-ADS setup with PMC. The model and compliance chamber were rotated 37° backwards to keep the device level during delivery when the curved Flex2 prongs were used (Fig.e 8). The inhalation breaths, which were ~0.3 and ~0.2 s for the full-term and preterm infant models, respectively, were delivered using the auto-actuator with the operator’s thumb over the exhalation port. A 1 – 2 s breath-hold was performed to simulate enhancement of deposition via particle sedimentation during an aerosol delivery scenario and prevent aerosol from being quickly exhaled due to the infant’s very high breathing rate. The operator’s thumb was then removed for exhalation and replaced once the pressure gauge read 4 – 6 cm H_2_O, to maintain PEEP. The next breath was then immediately delivered. Four breathing cycles with average durations of ~3.5 s are shown in Fig. 9 for each infant model. The PIPs (including dynamic pressure effects) were below the threshold of 25 cm H_2_O at ~19 and ~22 cm H_2_O for the full-term and preterm models, respectively (Table II). Fig. 9 shows some air leak, which was expected when adding the full NT model and iDP-ADS to the chamber. These small leaks were deemed acceptable since powder losses were not evident during experiments, the PIPs were consistent, and the PEEP could be reasonably applied.

During powder delivery, the pressure sensor at the PMC was removed to prevent harm and powder contamination. The PMC was sealed off, and the system was vented via a valve past the filter to keep powder from dislodging from the filter during exhalation. In both testing scenarios, the approximate lung pressure was maintained by watching the pressure sensor in the compliance chamber.

### Infant NT Airway Models

Two anatomically-correct NT models, a full-term and a 34-week preterm infant model, were used to test the performance of the iDP-ADS across the later GA patient population. The full-term model (Fig. 7a) was the same model described in Howe *et al*. [38] and scaled from the 6-month-old model in Bass *et al*. [69]. In summary, the face, nasal cavity, pharynx, and larynx were extracted from the computed tomography (CT) scan of a 6-month-old infant, then scaled to a target weight between 3.2 to 3.5 kg. The average boys’ and girls’ 50^th^-percentile height at an age of 0 months was taken from the World Health Organization (WHO) growth charts [70] for scaling. Height-ratio-based scaling has been shown to produce good agreement in terms of aerosol deposition between infants of different ages [71].

The 34-week (2.20 kg) GA preterm infant NT model (Fig. 7b) has recently been presented by Hasan *et al*. [41] as part of a preterm model series spanning the 28-week to 34-week GA preterm infant population. The 34-week GA model is a scaled version of the anatomy of two slightly larger infants: the nasal cavity through nasopharynx from a 2.7-kg male and the oropharynx through larynx of a 2.3-kg female infant. These regions were extracted from CT scans and smoothly combined before being isotropically scaled based on height to the 2.20-kg size. Dead space measurements were used to help verify anatomical correctness [41].

Both NT models were sectioned into Anterior, Middle, and Throat regions (Fig. 7c) to assess regional deposition during experiments. Each section was connected using twist locks with O-rings for sealing. The Middle and Throat for each model were printed by Quickparts (Seattle, WA) with an SLA printer using Accura ClearVue resin at high resolution. The Anterior nasal portions, including the faces and nares, were cast in silicone (Dragon Skin™; Smooth-On, Macungie, PA) to mimic the elasticity of skin. This flexible portion was affixed to a 3D-printed rigid face adaptor with connections for sturdy attachment to the Middle passage (Fig. 7d). The Throat ended in attachments for a LV filter housing. Both the Anterior rigid face adaptor and LV filter were built on a Stratasys Objet24 3D Printer (Stratasys Ltd., Eden Prairie, MN) using VeroWhitePlus resin and a resolution of 32 µm.

### Powder Materials and Formulation

Albuterol sulfate (AS) and l-leucine were purchased from Sigma-Aldrich Chemical Co. (St. Louis, MO). Poloxamer 188 was donated from BASF Corporation (Florham Park, NJ). Trileucine was purchased from Bachem Americas, Inc. (Torrance, CA). D-mannitol, sodium chloride, ethanol and methanol were purchased from Fisher Scientific Co. (Hanover Park, IL). Throughout the study, freshly collected deionized water was used.

Three albuterol sulfate excipient enhanced growth (AS-EEG) powder batches were prepared by using a Büchi Nano B-90 HP Spray Dryer (Büchi Laboratory-Techniques, Flawil, Switzerland) or a custom small-particle spray dryer described in Aladwani *et al*. [72], following the optimized method described by Son *et al*. [49]. Briefly, the feed solution of the formulation (composed of a 30:48:20:2% w/w ratio of AS, mannitol, l-leucine or trileucine, and Poloxamer 188, respectively) was prepared by dissolving the formulation in a water and ethanol (80:20% v/v) co-solvent system at a solids concentration of 0.5% w/v. A feed solution volume of 150 mL was sprayed at a spray rate between ~0.40 – 0.60 mL/min. The feed solution temperature was between 5 – 15°C during spray drying while the excess feed solution was recycled into the cooled stock. The spray-dried powders were collected from the electrostatic precipitator into glass vials and equilibrated for one week by keeping the vial lids open (in a desiccator, ~5% RH/~22°C) before using for experiments. After one week, the lids were closed and stored in the same desiccator when not in use.

Across the two available small-particle spray drying platforms, three individual batches of powder were prepared using the specified drying conditions. These three batches were then respectively assigned to the three major comparisons evaluated in this study:

- Batch 1 (trileucine-containing AS-EEG) for the comparison of flow pathways (Table III);
- Batch 2 (l-leucine-containing AS-EEG) for the evaluation of flexible prongs (Table IV); and
- Batch 3 (l-leucine-containing AS-EEG) for the impact of PM and the second NT model (Table V).

To compare designs and effects consistently, the previous best-performing setup was chosen and retested with the next powder batch. For example, the best-performing flow pathway from Table III using Batch 1 powder was retested with the Batch 2 powder to isolate the effect of the change in prong design.

Approximate characterizations of the AS-EEG formulation produced under similar spray drying conditions have been performed previously. Son *et al*. [49] performed scanning electron microscopy (SEM) analysis of leucine-containing AS-EEG formulations. Howe *et al*. [38] measured a geometric diameter of 0.99 (0.0) µm (equivalent to an MMAD of 1.17 µm, assuming a formulation density of 1.393 g/cm^3^) for an l-leucine-containing AS-EEG formulation using a Sympatec ASPIROS dry dispersing unit and HELOS laser diffraction sensor (Sympatec GmbH, Clausthal-Zellerfeld, Germany) under a pressure of 4 bar. In other work, identical primary particle size measurement conditions have resulted in MMADs of 1.18 – 1.20 µm for l-leucine-containing AS-EEG formulations [73, 74]. Howe *et al*. [40], using a Next Generation Impactor (NGI) (MSP, TSI Incorporated, Shoreview, MN), measured an MMAD of 1.56 µm for an AS-EEG aerosol (with l-leucine as the dispersion enhancer) exiting the same infant air-jet DPI used in this study at a Q90 flow rate of 1.7 LPM with the auto-actuator air source (D1-Single-Timer in Howe *et al*. [40]).

### Experimental Procedure

Phases 1 and 2 of the experiments had identical experimental procedures. First, the three sections of the NT model – Anterior, Middle, and Throat (Fig. 7c) – were coated with MOLYKOTE® 316 silicone spray (Dow Corning, Midland, MI) to prevent particle bounce and aerosol re-entrainment after depositing on the model. The actuation flow rate was set using the Q90 metric. To calibrate the Q90 value before each run for Phase 1, the device and interface were connected to the flow meter, and the inlet pressure into the auto-actuator (EM) timer was adjusted to achieve the desired Q90 value. The actuation time was set on the auto-actuator to deliver a nominal actuation volume of 30 mL based on calculations using the desired Q90. For Phase 2, the Q90 calibration was more rigorous. The actuation time was determined by ensuring the target actuation volume fell within the tolerances set for the tidal volume. Upon successful calibration, the aerosolization chamber was loaded with 10 mg of the AS-EEG formulation. Vacuum grease was added around the base of the prongs to ensure an airtight seal. Then, the interface was placed in the nostrils, while ensuring that the device was held horizontally. The timer was actuated three times with approximately 5 s between actuations. For the FP0 interface, the contralateral nostril had to be pinched shut during actuation and breath-hold then released before the next actuation. To quantify aerosol deposition after delivery, the device, interface, filter, and three parts of the NT model (Anterior, Middle, and Throat) were disassembled and washed in known amounts of deionized water until the formulation was fully dissolved. The solution was placed in vials and the albuterol concentrations were quantified using a validated high performance liquid chromatography (HPLC) method. Experimental data were reported in terms of recovered dose from the HPLC, and emitted doses were calculated based on total recovered dose minus the doses deposited in the device and interface (e.g., the dose exiting the patient interface and entering the NT model).

### Drug Mass Characterization and HPLC Analysis

A validated HPLC method was used to measure the content uniformity (*n* = 3, for each powder) of AS-EEG powder together with quantifying albuterol sulfate in each deposition region during aerosol performance testing. A Waters e2695 separations module with a 2475 fluorescence detector (excitation = 276 nm, emission = 609 nm) and Empower 3 data acquisition software (Waters Co., Milford, MA) was used. Chromatography was performed using a Restek Allure PFPP (5 µm, 60 Å, 150 x 2.1 mm) column (Bellefonte, PA) with a mobile phase flow rate of 0.4 mL/min. The mobile phase consisted of methanol and ammonium formate buffer (20 mM, pH 3.4) in a ratio of 70:30. The column temperature was maintained at 25°C, and the injection volume of each sample was 10 μL. Before sample analysis, a range of albuterol sulfate standards (0.5 – 20 µg/mL) were prepared using deionized water and injected as the calibration standard. All the samples (content uniformity and deposition samples) were also prepared by dissolving in an appropriate volume of deionized water.

### Statistical Analysis

At least three replicates were run for each experiment. Statistical analysis was performed using JMP Pro 17 (SAS Institute Inc., Cary, NC) and all statistical tests used a significance limit of $p=$ 0.05. For comparing the flow pathway designs and the prong designs, ANOVA tests were first conducted followed by Tukey’s post hoc. For comparisons between the filter and PM and between the full-term and preterm PM models, Student’s t-Tests were performed.

## Results

### Comparison of Flow Pathway Designs

Aerosol deliveries from the iDP-ADS through the full-term (3.55 kg) NT model and tracheal filter (without PM) for different flow pathways are reported in Table III and resulted in several key observations. First, direct comparison between FP0 and FP1 indicated that the addition of the bifurcation nearly doubled interface losses. However, due to the lower NT loss in the FP1 case, perhaps due to the reduced inertial impaction resulting from the reduced flow through two nostrils instead of one, the lung deliveries were nearly identical. It is worth noting that the middle nasal cavity deposition decreased by a statistically significant amount (~3% absolute difference) in all dual-outlet cases as compared to the single-prong delivery strategy. Second, the rapid expansion design of FP2 nearly doubled the interface deposition again compared to FP1. This rapid diffusion of the jet and boundary layer reattachment caused significant particle deposition, but it may have filtered out larger particles as well, given the statistically significant lower total NT loss. Third, the addition of the fins in the FP3 design again increased the interface losses compared to FP2. These fins provided greater area for particle deposition. Additionally, the NT losses were similar between FP2 and FP3, indicating that no additional benefits of particle filtering or flow straightening in the interface translated to improved lung delivery. In fact, FP3 was the only design to result in statistically lower filter deposition. Finally, the addition of co-flow in FP4 cut interface losses by more than half compared to FP2 (7.9% *vs*. 19.0%), which was the same design but without co-flow; therefore, it was the only interface not to have statistically different deposition compared to FP0. While device retention was higher in FP4 due to the reduced actuation volume used during aerosolization (30 mL *vs*. 10 mL), the overall effect was a high lung delivery efficiency of ~55%. Even with the higher total flow rate of 5.1 LPM, total NT losses were not statistically larger than in the FP0 case but were ~6% lower (absolute difference), indicating that there may have been some sheathing of the aerosol flow or an aerosol concentration effect even into the nasal cavity. FP4 (co-flow) was chosen for further testing due to its high filter delivery efficiency, which achieved the original objective of equivalent or greater filter delivery as compared to the single-outlet interface; low interface deposition; and potential for future optimization.

**Table III:** Comparison of Filter Delivery Efficiency in Terms of Recovered Dose for Different Bifurcating Flow Passages Testing the Full-Term Model with the Filter Open to the Atmosphere

Interface	FP0 ($n=5$)	FP1 ($n=3$)	FP2 ($n=4$)	FP3 ($n=4$)	FP4 ($n=7$)
Device (%)	10.9 (3.7)	10.2 (2.8)	10.2 (2.5)	11.7 (4.1)	15.4 (3.1)^*^
Interface (%)^a^	6.7 (1.5)	11.9 (1.1)^b^	19.0 (3.7)^b^	24.9 (3.0)^b^	7.9 (0.5)
Anterior (%)^a^	6.3 (1.7)	9.3 (0.5)	5.6 (0.4)	6.9 (2.1)	6.9 (1.1)
Middle (%)^a^	8.8 (3.3)	4.8 (0.2)^b^	4.7 (1.1)^b^	4.4 (0.6)^b^	5.1 (1.1)^b^
Throat (%)^a^	12.3 (1.9)	9.2 (2.0)	6.5 (1.3)^b^	5.7 (1.8)^b^	9.5 (2.0)
Total NT Loss (%)^a^	27.4 (5.8)	23.4 (2.5)	16.7 (1.1)^b^	17.1 (3.6)^b^	21.6 (3.0)
**Tracheal Filter (%)**^**a**^	**54.9 (2.6)**	**54.6 (0.6)**	**54.1 (3.4)**	**46.2 (2.8)**^**b**^	**55.2 (3.5)**
Emitted Dose (%)^a^	82.4 (4.7)	78.0 (2.4)	70.8 (4.1)^b^	63.4 (1.6)^b^	76.8 (2.9)
Q90 (LPM)^c^	1.68 (0.03)	2.00 (0.03)	1.98 (0.04)	2.01 (0.03)	5.26 (0.19)

Reported as mean (standard deviation)

^*^Includes deposition in both the device and the co-flow entry region before the interface

^a^Significant effect of interface on deposition region (*p* < 0.05, ANOVA)

^b^Significant difference compared with FP0 (*p* < 0.05, Tukey HSD)

^c^Flow rates for FP1, FP2, and FP3 were not significantly different amongst themselves ($p<0.05$, Tukey HSD) as expected. FP0 and FP4 were significantly different from all others

FP = Flow pathway, NT = Nose-throat

### Comparison of Prong Designs

Evaluation of different flexible prong designs showed that filter delivery performance remained unaffected by using a rigid conical design as compared to a flexible, more ergonomic design (Table IV). In the interface, Flex1 showed slightly, but statistically significant, higher losses compared to the Rigid Cone, perhaps due to the compression of the prong in the more flexible material. The more rigid Flex2 design did not show higher losses, even with the addition of the curvature in both the bifurcation and prongs. The anterior nasal cavity deposition was statistically lower for both flexible designs, indicating that Flex1 and Flex2 achieved better positioning and directing of the aerosol jet in the nose, at least initially. Total NT loss was unchanged. The incoming jet must have been redirected into the middle nasal cavity to some extent. Importantly, while the flexible designs did not improve filter delivery statistically, they did not adversely affect it either. The Flex2 design was thus chosen for future experiments since it was a highly-functional, simply-constructed, 3D-printed design for a wide-range of patient nasal geometries and did start an upward trend towards higher tracheal filter delivery.

**Table IV:** Comparison of the Filter Delivery Efficiency in Terms of Recovered Dose between the Original Rigid Cone Prongs and Two Flexible Prong Types Combined with the Best-Performing Flow Pathway, FP4 (Co-Flow Interface Design)

Prong	Rigid Cone ($n=3$)	Flex1 ($n=5$)	Flex2 ($n=3$)
Device (%)^*^	16.1 (0.3)	13.5 (3.0)	15.3 (2.5)
Interface (%)^a^	9.0 (0.4)	11.9 (1.0)^b^	9.3 (0.8)
Anterior (%)^a^	7.4 (0.5)	5.6 (1.1)^b^	4.7 (0.1)^b^
Middle (%)	6.1 (0.7)	7.9 (0.9)	8.1 (1.6)
Throat (%)	10.1 (0.4)	9.2 (1.1)	9.7 (1.0)
Total NT Loss (%)	23.5 (1.4)	22.7 (1.9)	22.6 (2.4)
**Tracheal Filter (%)**	**51.4 (0.7)**	**51.8 (3.2)**	**52.9 (4.3)**
Emitted Dose (%)	74.9 (0.6)	74.6 (2.7)	75.4 (2.1)
Q90 (LPM)	5.18 (0.12)	5.30 (0.04)	5.39 (0.29)

Reported as mean (standard deviation)

^*^Includes deposition in both the device and the co-flow entry region before the interface

^a^Significant effect of prong type on deposition region (*p* < 0.05, ANOVA)

^b^Significant difference compared with Rigid Cone (*p* < 0.05, Tukey HSD)

NT = Nose-throat

### Effects of Realistic Pulmonary Mechanics and Infant Age

Table V shows that there were statistically significant effects of infant age, but not downstream PM, on filter deposition. In the full-term setup, filter deposition decreased by ~4% (absolute difference) when PM were implemented. Although the difference between filter depositions in the filter-only and PM cases did not differ by the predetermined significance threshold of $p=$ 0.05, the p-value of 0.0527 sat very close to this value. This decrease may have been the effect of downstream pressures on the emitted dose. Although the Q90s between the filter-only and PM cases for the full-term infant model were significantly different (Table V), flow rate differences on the order of ~0.2 LPM did not significantly influence results, especially at these high flow rates. As expected, the filter delivery of the 34-week preterm infant model was statistically lower (~11% absolute difference) than the full-term infant model. Deposition was significantly higher in the interface, middle nasal cavity, and overall NT. The same interface, including prong size, was used for both infant models. Given the smaller actuation volume for the preterm infant (14.7 mL), there was a smaller ratio of actuation-volume-to-dead-space compared to the full-term infant model (23.7 mL actuation volume). Pressure effects may have also decreased the emitted dose. The measured PIP was ~22 cm H_2_O as compared to ~19 cm H_2_O in the full-term model. Additionally, the prongs fit more tightly into the smaller infant, perhaps leading to some compression in the prongs. This compression may explain a modest increase in the jet effect into the middle nasal cavity as indicated by the statistically higher deposition in this region. NT losses, though, were expected to be higher given the smaller size of the preterm airways. Interestingly, the lower AF actuation volume of 4.9 mL in the 34-week preterm scenario did not significantly affect device emptying as compared to the full-term case with an AF of 7.9 mL; therefore, a range of AF delivery volumes may produce consistent and high-quality powder aerosolization, which is needed in the highly-variable preterm patient population.

**Table V:** Evaluation of Filter Delivery Efficiency (% of Recovered Dose) With and Without PM for the Full-Term Infant Model and between the Full-Term and 34-Week Preterm Models with Compliance Chamber and Size-Appropriate PM

Model	Full-Term Filter Only ($n=3$)	Full-Term PM ($n=3$)	34-Week Preterm PM ($n=3$)
Device (%)^*^	19.9 (1.1)	21.6 (2.8)	23.1 (2.2)
Interface (%)	14.6 (0.1)	14.8 (1.2)	17.7 (1.2)^b^
Anterior (%)	4.2 (0.1)	3.7 (1.2)	6.8 (2.2)
Middle (%)	5.2 (0.6)	6.5 (1.7)	10.3 (0.9)^b^
Throat (%)	6.6 (1.0)	8.1 (2.0)	8.2 (1.1)
Total NT Loss (%)	16.0 (1.4)	18.3 (2.5)	25.3 (2.2)^b^
**Tracheal Filter (%)**	**49.4 (2.0)**	**45.4 (1.6)**	**33.9 (1.2)**^b^
Emitted Dose (%)	65.5 (1.2)	63.7 (1.7)	59.2 (1.0)^b^
Q90 (LPM)	5.28 (0.10)	5.04 (0.09)^a^	5.11 (0.07)

Reported as mean (standard deviation)

^*^Includes deposition in both the device and the co-flow entry region before the interface

^a^Significant difference between filter only and pulmonary mechanics setups for the full-term model (*p* < 0.05, Student’s t-Test)

^b^Significant difference between models (*p* < 0.05, Student’s t-Test)

PM = Pulmonary mechanics, NT = Nose-throat

## Discussion

This study focused primarily on improving the iDP-ADS through developing a bifurcation interface and prongs capable of high aerosol transmission and then testing this interface with a PMC in two neonatal NT models. A major finding was that a dual-outlet interface can achieve at least equivalent delivery compared to a single-prong approach by using a bifurcation geometry; therefore, a bifurcation interface can be used to simplify administration technique without reductions in aerosol delivery performance. Furthermore, it has applications for integration into or translation to a CPAP interface [41]. The bifurcation improved upon previous dual-outlet designs. Howe *et al*. [39] reported significant reductions in tracheal filter delivery (>10% absolute difference) when investigating a dual-outlet DPI with two independent gradual expansion regions attached to each DPI outlet. Interestingly, these losses mostly came from NT deposition in the dual-outlet configurations. These high deposition losses from Howe *et al*. [39] contradicted those predicted in this study, where total NT losses for all dual-outlet designs were lower (even if not significantly) compared to the single-outlet case. Similarly, in the study of Bass *et al*. [43], deposition efficiency curves in the preterm NT were lower when using dual-prong delivery as compared to single-prong delivery, likely due to the lower Stokes number when splitting the total flow rate through two prongs. Perhaps one reason for the higher NT losses in the dual-prong design of the Howe *et al*. [39] study might be particle growth across the interface, either through particle agglomeration and re-entrainment via stripping from the sides of the interface or some other mechanism. At a Q90 of 1.7 LPM, a larger NGI-measured MMAD at the outlet of the dual-outlet interface (2.63 µm) as compared to the single-outlet gradual expansion (FP0 in this study) interface (1.90 µm) was reported in Bass *et al*. [43]. These larger particle sizes for the dual-outlet interface were introduced into the nose, resulting in higher NT drug losses as compared to the single-outlet interface. The CF interface in this study kept the particles from depositing on the walls (as indicated by the reduction in interface loss), thereby preventing particle size increases through agglomeration across the interface. Even though the aerosol was entering the nose at a higher flow rate of 5.1 LPM, the smaller outlet particle diameter from the interface would keep the impaction parameter, and thus deposition, lower in the NT. Additionally, the single-outlet DPI may perform better than the dual-outlet DPI design at the lower flow rates of 1.7 LPM used in the Howe *et al*. interface study [39] as compared to 4 LPM, the only flow rate tested, which provided comparable aerosolization performance between single- and dual-outlet DPIs [40]. Regardless of the physical mechanism, the dual-prong strategy and dual prongs with co-flow resulted in lower NT losses and similar lung delivery compared to the single-prong interface.

Another finding of this study was that the Flex2 prongs provided a good fit and performed well in both models, indicating that only a few prong sizes may be necessary across the preterm to full-term infant population by leveraging this flexible bifurcation and wedge-shaped prong design. The iDP-ADS is being developed for infants >1 kg (~28 weeks GA; end of extremely preterm GA) to 3.55 kg (39 through 40 weeks GA; full-term), and the use of a single prong size in this study covered a 1.35-kg (5 – 6 weeks GA) range equivalent to approximately 40 – 50% of the target patient population. Furthermore, the two infant models evaluated had diverse nasal anatomy. The full-term model’s nostril shape was more elliptical with average major and minor diameters between left and right nostrils of ~6.1 mm and ~5.3 mm, respectively, and a septal distance of ~3.8 mm. The 34-week GA preterm model had more asymmetric nostrils with a septal distance of ~2.5 mm. Both nares had a rounded square shape, but the left nostril outline was skewed with a larger variation in minimum and maximum diameters across the diagonal of the “square” outline of ~5.6 mm and ~6.1 mm, respectively, as compared to those of ~5.9 mm and ~6.3 mm, respectively, for the right nostril. Thus, the current prong design accommodated dimensional differences and compensated for different morphologies. Consequently, the number of necessary prong sizes in a packaged kit intended to address a wide variety of preterm infant weights and sizes may need to include only 2 – 3 prong sizes (especially for future CPAP interface applications where nasal trauma is a primary concern) or 1 prong size with slight adjustments of the current prong design for a D2I approach. Current commercially available CPAP systems have a large number of interfaces from which to choose, with 10 to 11 options for the Dräger BabyFlow^®^ (Dräger) CPAP system and the Fisher & Paykel FlexiTrunk™ (Fisher & Paykel Healthcare) CPAP interface, respectively, to 3 to 4 options for the Argyle™ (Cardinal Health™) and Inspire nCPAP™ (Inspiration Healthcare Group PLC) CPAP interfaces, respectively. Fewer interface options would streamline manufacturing and reduce costs as well as simplify setup and administration in a clinical setting. Additionally, as compared to rigid prong designs, these more optimally-positioned and flexible prongs could help improve sealing at the nostrils and reduce nasal injury, especially in future CPAP applications with prolonged use.

The Flex2 prongs can be incorporated into other interfaces and aerosol delivery systems without reductions in lung delivery. Using the streamlining technique to minimize sharp turns [53, 54], the 45° bend of the bifurcation of Flex2 permits removal of the 37° bend of the device outlet capillary, which could simplify future manufacturing of the device, without increases in depositional losses (9.3% for Flex2 *vs*. 9.0% for Rigid Cone (Table IV)). The choice of higher shore hardness (80A) balanced the tradeoffs of the rigid, less versatile prongs and the softer (60A) prongs that were more easily compressed and led to higher deposition in the interface (11.9%). The jet effect in the nasal cavity from the compression of the Flex1 prongs, though, did not result in significantly different NT losses, potentially because the prongs were not inserted too deep into the nose (~7 mm) for a full-term infant; therefore, the jet was able to dissipate some before impaction in the nasal cavity. Additionally, the angled cut on the Flex1 prongs directed the aerosol downwards as evidenced by the significantly lower anterior deposition (5.6%) compared to the rigid prongs (7.4%). Similarly, the optimally angled positioning of Flex2 resulted in low anterior deposition (4.7%). This observation can be compared to the study of Bass *et al*. [43], who showed that external rigid prongs and compressible flexible prongs inserted 5 mm have similar NT losses in a preterm NT model. However, this jet effect resulted in markedly higher deposition at deep insertion depths of 11 mm. The use of angled cuts as in Flex1 and curved prong tips as in Flex2 could allow for a couple millimeters of deeper insertion. Sufficient insertion into the nose may be important for consistent alignment of the prongs in the nasal cavity and secure positioning of the interface during pressurization and aerosol delivery. However, it should be noted that shallower insertion may be necessary in younger preterm patients since their nasal cavities are smaller and jet effects may be more significant over these smaller lengths. In summary, the Flex2 prongs demonstrated a combination of features – proper material selection, streamlined flow passages, and insertion depth – that resulted in high, clinically relevant, delivered lung efficiencies (filter delivery of ~53%) and also inform future prong optimization studies.

With the best-performing interface, the filter deliveries of ~45% for the full-term infant and ~34% for the 34-week GA infant with PM (Table V) were high compared to previous non-invasive aerosol delivery systems not employing condensational growth technologies, which range from 0 – 14% lung delivery with high intersubject variabilities [11–13, 15, 16, 18, 75–77]. Many of these systems are nebulizer-based, which, as reviewed extensively elsewhere [78], have several features, such as high breathing circuit dead volumes and long therapy time requirements, that make aerosol delivery to infants difficult with their short inhalation times and low tidal volumes. With the D2I approach, the aerosol is delivered close to the patient and supplies the inhalation breath with the aerosol, which prevents aerosol loss during exhalation. Application of the PMC showed that a breathing maneuver to enhance aerosol deposition using a brief 1 – 2 s breath-hold can be applied consistently, thereby eliminating one factor of inter- and intrasubject variability, and within targeted pressure ranges. Due to these features and this delivery strategy, lung delivery efficiencies were high under realistic conditions.

Unlike previous work [40, 51], the downstream PM did affect deposition slightly (~4% reduction in filter deposition), although not significantly by a thin margin ($p=$ 0.0527), perhaps due to the higher flow rate with FP4 of 5.1 LPM used in this study and the resulting increased downstream dynamic pressures. There also may have been some effect on powder compression due to the downstream pressure and higher aerosol masses not fully clearing the NT, thus remaining suspended for longer. Although PM did not have a large effect on lung delivery, future designs may benefit from lower flow rates, which would reduce dynamic downstream pressures and potentially improve aerosol delivery.

The iDP-ADS addresses many of the shortcomings of existing aerosol delivery systems and strategies. However, additional improvements and rigorous testing are needed for a clinical-level system. First, the best-performing interface with CF must be tested with the treatment formulation – e.g., surfactant for RDS. Since different formulations have specific powder properties, the benefits of CF and the dual-outlet strategy may not be equivalent to the outcomes of the AS formulation in this study. Second, the CF design can be advanced by testing additional designs and optimizing the current system, especially for the lower GA preterm infant population, which has smaller tidal volumes and will need smaller versions of the interface. Third, additional techniques such as chase air, where the aerosol cloud is delivered towards the beginning of the inhalation and clean air is sent in following the aerosol cloud to clear the dead space of aerosol and push it into the lungs, could be implemented into current and future designs. The concept of delivering the aerosol during the first half of inhalation has been investigated in an intubated full-term infant model by Longest *et al*. [56]. They demonstrated that this strategy cleared most of the delivery components and endotracheal tube of the aerosol after the full inhalation, as compared to the approximately 30 – 40% of the aerosol suspended after delivering the aerosol over the full inhalation. As a result, the exhaled dose for half-inhalation aerosol delivery was approximately four times lower and the delivered lung dose, even with exhalation included, was doubled compared to full-inhalation aerosol delivery. For these scenarios and others, future whole-lung modeling is essential to verify their respective effects on regional lung targeting.

Rigorous safety testing across the applicable patient population will be imperative for approval in a clinical setting. Currently, animal testing in rabbits using the iDP-ADS, including pressure application with the PMC, to deliver a synthetic lung surfactant are ongoing and show promising efficacy and safety results [36], but more studies are needed. To protect the underdeveloped premature lung, pressure monitoring is critical; therefore, additional features, such as a pop-off valve at the interface, and strategies, such as ensuring that aerosol delivery is appropriately timed with inhalation, require exploration. An adaptation of the iDP-ADS for simultaneous aerosol delivery during nasal CPAP is also in development [41], thereby expanding the breadth of testing needed for safe aerosol delivery and ventilation. The best-performing flow pathway, prongs, and PMC from this study, though, in addition to the insights gained, can be translated to a future CPAP interface.

## Conclusions

Multiple bifurcating, dual-outlet interfaces matched deposition of the previously best-performing single-prong interface – a primary goal of this study – and achieved lung delivery efficiencies of ~55% of the nominal dose. The CF design (FP4) greatly reduced interface losses from a high of ~25% to ~8%, which are similar to losses in the single-outlet baseline interface. The inclusion of a flexible bifurcation and prongs with added curvature did not compromise the tracheal filter delivery. This flexible but firm prong design had several benefits, including the potential for reduced nasal lung injury due to the ergonomic design, improved positioning in the nostrils, and applicability to a range of infants from late-preterm to full-term. To improve the safety and consistency of aerosol administration, a PMC unit adequately monitored and controlled pressures during aerosol delivery, and all ventilation parameters remained within targeted and effective ranges. In conclusion, the iDP-ADS potentially addresses the needs for safe and efficient pharmaceutical aerosol delivery in the currently underserved infant patient population.

## Data Availability

The datasets generated and evaluated during the current study are not publicly available but are available from the corresponding author upon reasonable request.

## Abbreviations

3D: Three-dimensional
AF: Aerosol flow
ANOVA: Analysis of variance
AS: Albuterol sulfate
AST: Aerosol surfactant therapy
CF: Co-flow
CFD: Computational fluid dynamics
CPAP: Continuous positive airway pressure
CT: Computed tomography
D2I: Direct-to-infant
DPI: Dry powder inhaler
ED: Emitted dose
EM: Electromechanical
EEG: Excipient enhanced growth
FP: Flow pathway
GA: Gestational age
HPLC: High performance liquid chromatography
ID: Inner diameter
iDP-ADS: Infant air-jet dry powder aerosol delivery system
LV: Low volume
MDI: Metered dose inhaler
MMAD: Mass median aerodynamic diameter
NGI: Next Generation Impactor
NIV: Non-invasive ventilation
NT: Nose-throat
PEEP: Positive end-expiratory pressure
PIP: Positive inspiratory pressure
PM: Pulmonary mechanics
PMC: Pressure monitoring and control
RDS: Respiratory distress syndrome
RH: Relative humidity
SD: Standard deviation
SEM: Scanning electron microscopy
SLA: Stereolithography

## References

[1] Willson DF. Aerosolized surfactants, anti-inflammatory drugs, and analgesics. Respir Care. 2015;60(6):774–93. 26070574 10.4187/respcare.03579

[2] Hartling L, Fernandes RM, Bialy L, Milne A, Johnson D, Plint A, *et al*. Steroids and bronchodilators for acute bronchiolitis in the first two years of life: systematic review and meta-analysis. BMJ. 2011;342:d1714. 21471175 10.1136/bmj.d1714 PMC3071611

[3] Walsh BK, Betit P, Fink JB, Pereira LM, Arnold J. Characterization of ribavirin aerosol with small particle aerosol generator and vibrating mesh micropump aerosol technologies. Respir Care. 2016;61(5):577–85. 26932383 10.4187/respcare.04383

[4] Mazela J, Polin RA. Aerosol delivery to ventilated newborn infants: historical challenges and new directions. Eur J Pediatr. 2011;170:433–44. 20878336 10.1007/s00431-010-1292-6 PMC3059826

[5] Rubin BK. Pediatric aerosol therapy: New devices and new drugs. Respir Care. 2011;56(9):1411–21. 21944688 10.4187/respcare.01246

[6] Hickey AJ. Back to the future: inhaled drug products. J Pharm Sci. 2013;102(4):1165–72. 23381932 10.1002/jps.23465

[7] Fink JB. Delivery of inhaled drugs for infants and small children: a commentary on present and future needs. Clin Ther. 2012;34(11):S36–45. 23149011 10.1016/j.clinthera.2012.10.004

[8] Newth CJL, Clark A. In vitro performance of the small particle aerosol generator (SPAG-2). Pediatr Pulmonol. 1989;7(3):183–8. 2529473 10.1002/ppul.1950070313

[9] Griffin DE. Current progress in pulmonary delivery of measles vaccine. Expert Rev Vaccines. 2014;13(6):751–9. 24837839 10.1586/14760584.2014.915753

[10] Rubin BK, Fink JB. Aerosol therapy for children. Respir Care Clin N Am. 2001;7(2):175–213. 11517020 10.1016/s1078-5337(05)70030-7

[11] Corcoran TE, Saville A, Adams PS, Johnston DJ, Czachowski MR, Domnina YA, *et al*. Deposition studies of aerosol delivery by nasal cannula to infants. Pediatr Pulmonol. 2019;54(8):1319–25. 30932345 10.1002/ppul.24326

[12] Sunbul F, Fink JB, Harwood R, Sheard MM, Zimmerman RD, Ari A. Comparison of HFNC, bubble CPAP and SiPAP on aerosol delviery in neonates: An in-vitro study. Pediatr Pulmonol. 2014. 10.1002/ppul.23123. 25491434 10.1002/ppul.23123

[13] El Taoum KK, Xi J, Kim J, Berlinski A. In vitro evaluation of aerosols delivered via the nasal route. Respir Care. 2015;60(7):1015–25. 25587167 10.4187/respcare.03606

[14] Réminiac F, Vecellio L, Loughlin RM, Le Pennec D, Cabrera M, Vourc’h NH, *et al*. Nasal high flow nebulization in infants and toddlers: an in vitro and in vivo scintigraphic study. Pediatr Pulmonol. 2017;52(3):337–44. 27392199 10.1002/ppul.23509

[15] Fok TF, Monkman S, Dolovich M, Gray S, Coates G, Paes B, *et al*. Efficiency of aerosol medication delivery from a metered dose inhaler versus jet nebulizer in infants with bronchopulmonary dysplasia. Pediatr Pulmonol. 1996;21(5):301–9. 8726155 10.1002/(SICI)1099-0496(199605)21:5<301::AID-PPUL5>3.0.CO;2-P

[16] Chua H, Collis G, Newbury A, Chan K, Bower G, Sly P, *et al*. The influence of age on aerosol deposition in children with cystic fibrosis. Eur Respir J. 1994;7(12):2185–91. 7713202 10.1183/09031936.94.07122185

[17] Sood BG, Thomas R, Delaney-Black V, Xin Y, Sharma A, Chen X. Aerosolized Beractant in neonatal respiratory distress syndrome: a randomized fixed-dose parallel-arm phase II trial. Pulm Pharmacol Ther. 2021;66:101986. 33338661 10.1016/j.pupt.2020.101986

[18] Bianco F, Ricci F, Catozzi C, Murgia X, Schlun M, Bucholski A, *et al*. From bench to bedside: in vitro and in vivo evaluation of a neonate-focused nebulized surfactant delivery strategy. Respir Res. 2019;20(1):134. 31266508 10.1186/s12931-019-1096-9 PMC6604359

[19] Longest PW, Farkas DR, Bass K, Howe C, Boc ST, Momin MAM, *et al*. Reviving positive pressure DPIs for efficient and reproducible aerosol delivery to infants and children. Respir Drug Deliv. 2020;1:71–80.

[20] LiCalsi C, Christensen T, Bennett JV, Phillips E, Witham C. Dry powder inhalation as a potential delivery method for vaccines. Vaccine. 1999;17(13–14):1796–803. 10194842 10.1016/s0264-410x(98)00438-1

[21] Farkas D, Hindle M, Longest PW. Development of an inline dry powder inhaler that requires low air volume. J Aerosol Med Pulm Drug Deliv. 2018;31(4):255–65. 29261454 10.1089/jamp.2017.1424 PMC6067687

[22] Islam N, Cleary MJ. Developing an efficient and reliable dry powder inhaler for pulmonary drug delivery - a review for multidisciplinary researchers. Med Eng Phys. 2012;34:409–27. 22277307 10.1016/j.medengphy.2011.12.025

[23] Farkas D, Hindle M, Longest PW. Application of an inline dry powder inhaler to deliver high dose pharmaceutical aerosols during low flow nasal cannula therapy. Int J Pharm. 2018;546(1–2):1–9. 29733972 10.1016/j.ijpharm.2018.05.011 PMC7253153

[24] Farkas D, Hindle M, Longest PW. Efficient nose-to-lung aerosol delivery with an inline DPI requiring low actuation air volume. Pharm Res. 2018;35(10):194. 30132207 10.1007/s11095-018-2473-7 PMC7253151

[25] Son Y-J, Longest PW, Tian G, Hindle M. Evaluation and modification of commercial dry powder inhalers for the aerosolization of submicrometer excipient enhanced growth (EEG) formulation. Eur J Pharm Sci. 2013;49:390–9. 23608613 10.1016/j.ejps.2013.04.011 PMC3744372

[26] Tian G, Longest PW, Li X, Hindle M. Targeting aerosol deposition to and within the lung airways using excipient enhanced growth. J Aerosol Med Pulm Drug Deliv. 2013;26(5):248–65. 23286828 10.1089/jamp.2012.0997 PMC3826577

[27] Delvadia R, Wei X, Longest PW, Venitz J, Byron PR. In vitro tests for aerosol deposition. IV: Simulating variations in human breath profiles for realistic DPI testing. J Aerosol Med Pulm Drug Deliv. 2015. 10.1089/jamp.2015.1215. 10.1089/jamp.2015.1215 PMC5079450 26447531

[28] Fuchs O, Latzin P, Thamrin C, Stern G, Frischknecht P, Singer F, *et al*. Normative data for lung function and exhaled nitric oxide in unsedated healthy infants. Eur Respir J. 2011;37(5):1208–16. 21109556 10.1183/09031936.00125510

[29] Estol P, Piriz H, Pintos L, Nieto F, Simini F. Assessment of pulmonary dynamics in normal newborns: a pneumotachographic method. J Perinat Med. 1988;16(3):183–92. 3062157 10.1515/jpme.1988.16.3.183

[30] Laube BL, Sharpless G, Shermer C, Sullivan V, Powell K. Deposition of dry powder generated by solovent in Sophia Anatomical infant nose-throat (SAINT) model. Aerosol Sci Technol. 2012;46:514–20.

[31] Walther FJ, Waring AJ, Otieno M, DiBlasi RM. Efficacy, dose–response, and aerosol delivery of dry powder synthetic lung surfactant treatment in surfactant-deficient rabbits and premature lambs. Respir Res. 2022;23(1):1–16. 35379243 10.1186/s12931-022-02007-8 PMC8978426

[32] Longest W, Farkas D. Development of a new inhaler for high-efficiency dispersion of spray-dried powders using Computational Fluid Dynamics (CFD) modeling. AAPS J. 2019;21(2):25. 30734133 10.1208/s12248-018-0281-y PMC7276205

[33] Boc S, Momin MA, Farkas DR, Longest W, Hindle M. Performance of Low Air Volume Dry Powder Inhalers (LV-DPI) when aerosolizing Excipient Enhanced Growth (EEG) surfactant powder formulations. AAPS PharmSciTech. 2021;22(4):1–12. 10.1208/s12249-021-01998-9 PMC8268434 33860378

[34] Momin MAM, Farkas D, Hindle M, Hall F, Diblasi R, Longest W. Development of a new dry powder aerosol synthetic lung surfactant product for neonatal Respiratory Distress Syndrome (RDS) - Part I: in vitro testing and characterization. Pharm Res. 2024;41(8):1703–23. 39112775 10.1007/s11095-024-03740-z PMC11362531

[35] Bass K, Farkas D, Hassan A, Bonasera S, Hindle M, Longest W. High-efficiency dry powder aerosol delivery to children: review and application of new technologies. J Aerosol Sci. 2021;153:105692. 33716317 10.1016/j.jaerosci.2020.105692 PMC7945982

[36] DiBlasi RM, KenKnight H, Kontoudios N, Farkas D, Momin MAM, Hall F, *et al*. Development of a new dry powder aerosol synthetic lung surfactant product for neonatal Respiratory Distress Syndrome (RDS) - Part II: in vivo efficacy testing in a rabbit surfactant washout model. Pharm Res. 2024;41(9):1827–42. 39237797 10.1007/s11095-024-03754-7 PMC11436456

[37] Rodenstein DO, Perlmutter N, Stanescu DC. Infants are not obligatory nasal breathers. Am Rev Respir Dis. 1985;131(3):343–7. 3977172 10.1164/arrd.1985.131.3.343

[38] Howe C, Hindle M, Bonasera S, Rani V, Longest PW. Initial development of an air-jet dry powder inhaler for rapid delivery of pharmaceutical aerosols to infants. J Aerosol Med Pulm Drug Deliv. 2021;34(1):57–70. 32758026 10.1089/jamp.2020.1604 PMC8182481

[39] Howe C, Momin MA, Bass K, Aladwani G, Bonasera S, Hindle M, *et al*. In vitro analysis of nasal interface options for high-efficiency aerosol administration to preterm infants. J Aerosol Med Pulm Drug Deliv. 2022;35(4):196–211. 35166601 10.1089/jamp.2021.0057 PMC9416545

[40] Howe C, Momin MA, Farkas DR, Bonasera S, Hindle M, Longest P. Advancement of the infant air-jet Dry Powder Inhaler (DPI): evaluation of different positive-pressure air sources and flow rates. Pharm Res. 2021;38(9):1615–32. 34462876 10.1007/s11095-021-03094-w PMC8642819

[41] Jubaer H, Strickler S, Farkas D, Dalton C, Momin MAM, Dodson K, *et al*. Development of CPAP overlay interfaces for efficient administration of aerosol surfactant therapy to preterm infants. AAPS PharmSciTech. 2024;26(1):34. 10.1208/s12249-024-02987-4. 10.1208/s12249-024-02987-4 39821052

[42] Longest W, Farkas D, Bass K, Hindle M. Use of Computational Fluid Dynamics (CFD) dispersion parameters in the development of a new DPI actuated with low air volumes. Pharm Res. 2019;36(8):110. 31139939 10.1007/s11095-019-2644-1 PMC7324281

[43] Bass K, Momin MA, Howe C, Aladwani G, Strickler S, Kolanjiyil AV, *et al*. Characterizing the effects of nasal prong interfaces on aerosol deposition in a preterm infant nasal model. AAPS PharmSciTech. 2022;23(5):1–18. 10.1208/s12249-022-02259-z 35441324

[44] Howe C, Momin MA, Aladwani G, Hindle M, Longest P. Development of a high-dose infant air-jet Dry Powder Inhaler (DPI) with passive cyclic loading of the formulation. Pharm Res. 2022;39:3317–30. 36253630 10.1007/s11095-022-03409-5 PMC10561662

[45] Tavernini S, Church TK, Lewis DA, Noga M, Martin AR, Finlay WH. Deposition of micrometer-sized aerosol particles in neonatal nasal airway replicas. Aerosol Sci Technol. 2018;52(4):407–19.

[46] Golshahi L, Noga ML, Finlay WH. Deposition of inhaled micrometer-sized particles in oropharyngeal airway replicas of children at constant flow rates. J Aerosol Sci. 2012;49:21–31.

[47] te Pas AB, Walther FJ. A randomized, controlled trial of delivery-room respiratory management in very preterm infants. Pediatrics. 2007;120(2):322–9. 17671058 10.1542/peds.2007-0114

[48] Dani C, Talosi G, Piccinno A, Ginocchio VM, Balla G, Lavizzari A, *et al*. A randomized, controlled trial to investigate the efficacy of nebulized poractant alfa in premature babies with respiratory distress syndrome. J Pediatr. 2022;246(40–7):e5. 10.1016/j.jpeds.2022.02.054 35257740

[49] Son Y-J, Longest PW, Hindle M. Aerosolization characteristics of dry powder inhaler formulations for the excipient enhanced growth (EEG) application: effect of spray drying process conditions on aerosol performance. Int J Pharm. 2013;443:137–45. 23313343 10.1016/j.ijpharm.2013.01.003 PMC3584634

[50] Walsh BK, Daigle B, DiBlasi RM, Restrepo RD. AARC clinical practice guideline. Surfactant replacement therapy: 2013. Respir Care. 2013;58(2):367–75. 23359726 10.4187/respcare.02189

[51] Howe C, Momin MAM, Aladwani G, Strickler S, Hindle M, Longest W. Advancement of a high-dose infant air-jet dry powder inhaler (DPI) with passive cyclic loading: Performance tuning for different formulations. Int J Pharm. 2023;643:123199. 37406945 10.1016/j.ijpharm.2023.123199 PMC10530264

[52] O’Donnell CP, Davis PG, Morley CJ. Resuscitation of premature infants: what are we doing wrong and can we do better? Biol Neonate. 2003;84(1):76–82. 12890941 10.1159/000071008

[53] Longest PW, Golshahi L, Hindle M. Improving pharmaceutical aerosol delivery during noninvasive ventilation: effects of streamlined components. Ann Biomed Eng. 2013;41(6):1217–32. 23423706 10.1007/s10439-013-0759-9 PMC3647043

[54] Longest PW, Azimi M, Golshahi L, Hindle M. Improving aerosol drug delivery during invasive mechanical ventilation with redesigned components. Respir Care. 2014;59(5):686–98. 24106320 10.4187/respcare.02782

[55] Bass K, Longest PW. Development of DPI patient interfaces for improved aerosol delivery to children. AAPS PharmSciTech. 2020;21:157. 32451773 10.1208/s12249-020-01667-3 PMC8662567

[56] Longest PW, Azimi M, Hindle M. Optimal delivery of aerosols to infants during mechanical ventilation. J Aerosol Med Pulm Drug Deliv. 2014;27(5):371–85. 24299500 10.1089/jamp.2013.1077 PMC4227441

[57] Dreyfuss D, Saumon G. Barotrauma is volutrauma, but which volume is the one responsible. Intensive Care Med. 1992;18(3):139–41. 1644960 10.1007/BF01709236

[58] Lista G, Castoldi F, Fontana P, Reali R, Reggiani A, Bianchi S, *et al*. Lung inflammation in preterm infants with respiratory distress syndrome: Effects of ventilation with different tidal volumes. Pediatr Pulmonol. 2006;41(4):357–63. 16477653 10.1002/ppul.20363

[59] Slutsky AS. Lung injury caused by mechanical ventilation. Chest. 1999;116(1 Suppl):9S-15S. 10424561 10.1378/chest.116.suppl_1.9s-a

[60] Gardner SL, Enzman-Hines M, Nyp M. Chapter 23: Respiratory Diseases. In: Gardner SL, Carter BS, Ensman-Hines MI, Niermeyer S, editors. Merenstein & Gardner’s Gandbook of Neonatal Intensive Care Nursing: An Interprofessional Approach. 9th ed. St. Louis: Elsevier, Inc.; 2020. p. 729–835.

[61] Sweet DG, Carnielli V, Greisen G, Hallman M, Ozek E, Te Pas A, *et al*. European Consensus Guidelines on the Management of Respiratory Distress Syndrome - 2019 Update. Neonatology. 2019;115(4):432–50. 30974433 10.1159/000499361 PMC6604659

[62] Walsh BK. Chapter 15: Noninvasive Mechanical Ventilation and Continuous Positive Pressure of the Neonate. In: Walsh BK, editor. Neonatal and pediatric respiratory care. 5th ed. St. Louis: Elsevier; 2019. p. 267–86.

[63] Bhutani VK, Bowen FW, Sivieri EM. Postnatal changes in pulmonary mechanics and energetics of infants with respiratory distress syndrome following surfactant treatment. Neonatology. 2005;87(4):323–31. 10.1159/000084880 15985755

[64] Tana M, Tirone C, Aurilia C, Lio A, Paladini A, Fattore S, *et al*. Respiratory management of the preterm infant: supporting evidence-based practice at the bedside. Children (Basel). 2023;10(3):535. 36980093 10.3390/children10030535 PMC10047523

[65] Schmolzer GM, Te Pas AB, Davis PG, Morley CJ. Reducing lung injury during neonatal resuscitation of preterm infants. J Pediatr. 2008;153(6):741–5. 19014815 10.1016/j.jpeds.2008.08.016

[66] Pandit PB, Pyon KH, Courtney SE, England SE, Habib RH. Lung resistance and elastance in spontaneously breathing preterm infants: effects of breathing pattern and demographics. J Appl Physiol (1985). 2000;88(3):997–1005. 10710396 10.1152/jappl.2000.88.3.997

[67] Mccann EM, Goldman SL, Brady JP. Pulmonary-function in the sick newborn-infant. Pediatr Res. 1987;21(4):313–25. 3574984 10.1203/00006450-198704000-00001

[68] Choukroun ML, Tayara N, Fayon M, Demarquez JL. Early respiratory system mechanics and the prediction of chronic lung disease in ventilated preterm neonates requiring surfactant treatment. Biol Neonate. 2003;83(1):30–5. 12566681 10.1159/000067015

[69] Bass K, Boc S, Hindle M, Dodson K, Longest W. High-efficiency nose-to-lung aerosol delivery in an infant: development of a validated computational fluid dynamics method. J Aerosol Med Pulm Drug Deliv. 2019;32(3):132–48. 30556777 10.1089/jamp.2018.1490 PMC6622559

[70] World Health Organization. WHO Child Growth Standards: Length/height-for-age, weight-for-age, weight-for-length, weight-for-height and body mass index-for-age: Methods and development. Geneva, Switzerland;2006.

[71] Tavernini S, Church TK, Lewis DA, Martin AR, Finlay WH. Scaling an idealized infant nasal airway geometry to mimic inertial filtration of neonatal nasal airways. J Aerosol Sci. 2018;118:14–21.

[72] Aladwani G, Momin MAM, Spence B, Farkas DR, Bonasera S, Hassan A, *et al*. Effects of different mesh nebulizer sources on the dispersion of powder formulations produced with a new small-particle spray dryer. Int J Pharm. 2023;642:123138. 37307962 10.1016/j.ijpharm.2023.123138 PMC10527815

[73] Farkas D, Bonasera S, Bass K, Hindle M, Longest PW. Advancement of a positive-pressure dry powder inhaler for children: use of a vertical aerosolization chamber and three-dimensional rod array interface. Pharm Res. 2020;37(9):177. 32862295 10.1007/s11095-020-02889-7 PMC8662578

[74] Farkas D, Hindle M, Bonasera S, Bass K, Longest W. Development of an inline dry powder inhaler for oral or trans-nasal aerosol administration to children. J Aerosol Med Pulm Drug Deliv. 2020;33(2):83–98. 31464559 10.1089/jamp.2019.1540 PMC7133455

[75] Reminiac F, Vecellio L, Heuze-Vourc’h N, Petitcollin A, Respaud R, Cabrera M, *et al*. Aerosol therapy in adults receiving high flow nasal cannula oxygen therapy. J Aerosol Med Pulm Drug Deliv. 2016. 10.1089/jamp.2015.1219. 26196740 10.1089/jamp.2015.1219

[76] Amirav I, Balanov I, Gorenberg M, Luder AS, Newhouse MT, Groshar D. Beta-agonist aerosol distribution in respiratory syncytial virus bronchiolitis in infants. J Nucl Med. 2002;43(4):487–91. 11937592

[77] Mallol J, Rattray S, Walker G, Cook D, Robertson CF. Aerosol deposition in infants with cystic fibrosis. Pediatr Pulmonol. 1996;21(5):276–81. 8726152 10.1002/(SICI)1099-0496(199605)21:5<276::AID-PPUL2>3.0.CO;2-L

[78] Bianco F, Salomone F, Milesi I, Murgia X, Bonelli S, Pasini E, *et al*. Aerosol drug delivery to spontaneously-breathing preterm neonates: lessons learned. Respir Res. 2021;22(1):1–31. 33637075 10.1186/s12931-020-01585-9 PMC7908012

